# Testicular germ cell tumour risk by occupation and industry: a French case–control study – TESTIS

**DOI:** 10.1136/oemed-2022-108601

**Published:** 2023-05-25

**Authors:** Margot Guth, Astrid Coste, Marie Lefevre, Floriane Deygas, Aurélie Danjou, Shukrullah Ahmadi, Brigitte Dananché, Olivia Pérol, Helen Boyle, Joachim Schüz, Louis Bujan, Catherine Metzler-Guillemain, Sandrine Giscard d’Estaing, Marius Teletin, Berengere Ducrocq, Cynthia Frapsauce, Ann Olsson, Barbara Charbotel, Béatrice Fervers

**Affiliations:** 1 UMRESTTE, Université Claude Bernard Lyon 1, Lyon, France; 2 Radiation: Defense, Health, Environment, INSERM UMR1296, Lyon, France; 3 Prevention Cancer Environnement Departement, Centre Léon Bérard, Lyon, France; 4 Environment and Lifestyle Epidemiology Branch, International Agency for Research on Cancer/World Health Organization (IARC/WHO), Lyon, France; 5 Department of Medical Oncology, Centre Léon Bérard, Lyon, France; 6 DEFE (Développement Embryonnaire, Fertilité, Environnement) INSERM 1202 Universités Montpellier et Toulouse 3, CECOS Hôpital Paule de Viguier, CHU de Toulouse, Toulouse, France; 7 Fédération Française des CECOS, Paris, France; 8 Centre Clinico-Biologique d’AMP-CECOS, AP-HM La Conception University Hospital, Marseille, France; 9 CECOS de Lyon, Service de Médecine de la Reproduction, Hôpital Femme Mère Enfant, Bron, France; 10 Department of Functional Genomics and Cancer, Institut de Génétique et de Biologie Moléculaire et Cellulaire, Illkirch-Grafenstaden, France; 11 CECOS Nord Lille, Hôpital Albert Calmette, Lille, France; 12 Médecine et Biologie de la Reproduction-CECOS, CHU Bretonneau, Tours, France; 13 Service des Maladies Professionnelles, Hospices Civils de Lyon, Pierre Bénite, France

**Keywords:** Epidemiology, Occupational Health, Risk assessment, Medical Oncology

## Abstract

**Objective:**

Testicular germ cell tumours (TGCT) are the most common cancer in men of working age and its incidence has increased notably over the past 40 years. Several occupations have been identified as potentially associated with TGCT risk. The aim of this study was to further explore the relationship between occupations, industries and TGCT risk in men aged 18–45 years.

**Methods:**

The TESTIS study is a multicenter case–control study conducted between January 2015 and April 2018 in 20 of 23 university hospital centers in metropolitan France. A total of 454 TGCT cases and 670 controls were included. Full job histories were collected. Occupations were coded according to the International Standard Classification of Occupation 1968 version (ISCO-1968) and industry according to the 1999 version of Nomenclature d’Activités Française (NAF-1999). For each job held, ORs and 95% CIs were estimated using conditional logistic regression.

**Results:**

A positive association was observed between TGCT and occupation as agricultural, animal husbandry worker (ISCO: 6–2; OR 1.71; 95% CI (1.02 to 2.82)), as well as salesman (ISCO: 4–51; OR 1.84; 95% CI (1.20 to 2.82)). An increased risk was further observed among electrical fitters and related, electrical and electronics workers employed for 2 years or more (ISCO: 8–5; OR_≥2 years_ 1.83; 95% CI (1.01 to 3.32)). Analyses by industry supported these findings.

**Conclusions:**

Our findings suggest that agricultural, electrical and electronics workers, and salesmen workers experience an increased risk of TGCT. Further research is needed to identify the agents or chemicals in these high-risk occupations which are relevant in the TGCT development.

**Trial registration number:**

NCT02109926.

**WHAT IS ALREADY KNOWN ON THIS TOPIC**

- Current evidence has not allowed to identify consistent occupational risk factors for testicular germ cell tumours (TGCT).
- In the past, several occupations and industries have been suggested to increase the risk of TGCT, including agricultural workers, firemen, policemen, military personnel and industrial workers (paper, electric, plastic or metal).
- Although TGCT incidence has increased further, only few studies have been published in recent years, while occupational health regulations may have lowered workplace exposures.

**WHAT THIS STUDY ADDS**

- Consistent with previous studies, an increased risk of TGCT among agricultural, electrical and electronics, and salesmen workers was observed.
- This observation suggests consistent associations over time of occupations with the risk of TGCT.

**HOW THIS STUDY MIGHT AFFECT RESEARCH, PRACTICE OR POLICY**

- The findings of this study offer important insights into the possible role of some high-risk occupations in the development of TGCT, suggesting the need to reinforce work health and safety measures.
- Further studies are needed to investigate the agents involved in the high-risk occupations identified in our study and assess their association with the risk of TGCT.

## Introduction

Testicular germ cell tumours (TGCT) are a rare cancer, but the most common cancer affecting men of working age (15–44 years) with an increasing incidence over the past 40 years, particularly among Caucasian populations.^1 2^ TGCT account for approximately 98% of malignant testicular tumours in France and are classified into two main histological subtypes, namely seminoma and non-seminoma.^3^

Genetic predisposition, personal and family history of TGCT are established as risk factors for TGCT.^2^ Differing incidence rates between population groups and geographical regions,^4^ and evolving incidence in migrant populations^5^ suggest a multifactorial origin, with complex interactions between genetic predisposition and yet unknown environmental factors.^3^ No specific environmental exposures have yet been identified, but endocrine disrupting chemicals (EDCs) are strongly suspected to play a role in TGCT development, and have been associated with increased incidence.^3 6^ Some organochlorines pesticides as well as polychlorinated biphenyls, hexavalent chromium and perfluorooctanoic acid (PFOA) have been associated with TGCT.^7–11^

Exposure to EDCs or carcinogens can occur in the workplace and the careful study of related occupations could lead to new hypotheses. The literature suggests a higher risk in certain occupations, such as agricultural, metal, electrical, construction, firemen, salesmen and clerical workers.^2 7 12^ However, most studies were published more than 10 years ago.^7 12^ Recently, the International Agency for Research on Cancer classified occupational exposure as a firefighter as ‘carcinogenic to humans’, with ‘limited’ evidence for testicular cancers.^13^ Occupational health regulations and preventive measures over the last decades may have changed some occupational exposures. For example, a decrease in the prevalence of French workers exposed to at least one carcinogenic, mutagenic and reprotoxic agents was observed from 13.7% to 10.6% in 2003–2010, in a context of tightened European regulations.^14^ Nevertheless, TGCT in Europe will further increase, particularly in historically low-risk countries.^15^ It is thus relevant to obtain current data to examine potential evolutions of occupation-TGCT associations over time to guide future interventions.

Therefore, we report the results of a French nationwide case–control study that investigated whether participants’ occupational history, by occupation and industries, may increase TGCT risk, overall and stratified by histological subtype. This study focuses specifically on TGCT occurring in young men, derived from germ cell neoplasia in situ (GCNIS) according to the 2016 WHO classification.^16^ Research hypotheses are distinct from that of non-GCNIS-derived tumours.^16^

## Methods

### Study design

The TESTIS study is a multicenter case–control study conducted between 2015 and 2018, in 20 out of the 23 university hospital (UH) in metropolitan France.^17^ TESTIS included patients (aged 18–45 years) with histologically confirmed TGCT, referred to a Centre d'étude et de conservation d'œufs et de sperme humain (CECOS) for pretreatment sperm cryopreservation. Recruitment with rapid ascertainment of cases through the CECOS was the most promising design resulting from the pilot study, to increase participation rates.^18^ Two control groups with no personal history of testicular cancer or cryptorchidism were also recruited: group A—sperm donors and partners of women consulting for fertility disorders, with normal sperm production, in CECOS ; group B—partners of women treated for a pathological pregnancy in specialised maternity clinics, adjacent to the CECOS. TGCT cases were individually matched to a control A and a control B on the recruiting UH and year of birth (±3 years).^17^ The target population (ie, young adult men) is particularly associated with low response rates,^19 20^ making it difficult to obtain an unbiased random sample of general population controls.^17^ In absence of a perfect control group, we chose two control groups presenting different aspects of the general population, as suggested by Stang *et al*.^21^ Moreover, according to the ‘testicular dysgenesis syndrome’ hypothesis, TGCT, cryptorchidism, hypospadias and some fertility disorders are presumed to originate from common dysfunction in intrauterine genital development.^4^ By selecting controls assumed to be fertile (group B) or with normal sperm count (group A), and without history of cryptorchidism, the risk of recruiting controls with minor forms of this syndrome was reduced. Regional catchment populations were similar for cases and both control groups.^17^ Participants provided written informed consent prior to study entry.

### Data collection

Of the 1323 participants included and having agreed to participate, 1124 subjects were interviewed by trained interviewers (IPSOS Company) blinded to the participants’ case–control status. The telephone interview used a structured questionnaire (85% response rate; cases: 87%; group A: 87%; group B controls: 79%). A detailed lifetime work history was recorded of all jobs and internships (>6 months): job title, industry, primary and secondary tasks performed, jobs’ start and end dates, compagnies’ name, address and activity, number of employees, and percentage of time worked. Participants received a document to prepare information on job history prior to the phone interview.^17^

### Ascertainment of GCNIS-related TGCT cases

Ascertainment of GCNIS-related TGCT cases has been detailed elsewhere.^17^ Briefly, cases were classified into seminoma and non-seminoma by a TGCT expert (HB). Given the low proportion of false-positive TGCT (5.1%), TGCT with missing pathology report (N=43) were included as cases.

### Study population

Overall, 1463 eligible subjects were invited to participate in the study, and 1367 (93.4%) agreed to participate. The population structure and reasons for non-participation were detailed previously.^17^ Of the 550 recruited cases, 96 were excluded: 21 non-GCNIS TGCT, 4 tumours not confirmed by pathology reports, 4 time from diagnosis to study inclusion >12 months, 1 missing date of diagnosis and 66 incomplete interviews (online supplemental figure S1). Of the controls, 9 not born in ‘metropolitan France’ (ie, excludes French subjects born in the French overseas territories), 5 with personal history of cryptorchidism and 133 with an incomplete interview were excluded. Finally, 1124 participants were included in the analyses.

10.1136/oemed-2022-108601.supp1 Supplementary data

### Coding of job titles

For each job, the occupation was coded according to the International Standard Classification of Occupation 1968 version (ISCO-1968) and industry according to the Nomenclature d’activités française 1999 version (NAF-1999). All coding was based on participants’ job title and task descriptions, blind to their case–control status. Some codes did not have the maximum number of digits (5 for ISCO, 4 for NAF) due to lack of information reported and were considered as aggregate codes. Aggregated codes were used in the code analyses up to their last known digit level and considered as missing for other levels.

### Covariates

Based on the literature, covariates established or suspected to be risk factors for TGCT^22 23^ were considered for adjustment (presented in table 1). These included perinatal factors: birth weight, gestational age, birth order, sibship size and born from multiple pregnancy. Further covariates included family history of testicular cancer; family history of cryptorchidism; personal tobacco smoking; cannabis use;^24^ and personal consumption of alcoholic beverages.^25^ We considered cannabis use and frequency of use in two periods: adolescence (12–17 years) and young adulthood (18–25 years), puberty being possibly considered as the key point of exposure.^24^ We used age at voice change as a proxy of timing of pubertal development to consider delayed puberty, a protective factor for TGCT.^26^ Although biologically questionable, TGCT risk has been reported in several studies to be associated with a personal history of testicular trauma.^23^ The selection of covariates followed a two-step process: first, we identified variables associated with TGCT in univariate analyses and selected those with p<0.20. All selected covariates were then included in one single regression model, and a manual backward stepwise selection procedure was performed. The final model included the following variables significantly associated with TGCT (p<0.05): sibship size, being born from multiple pregnancy, personal history of testicular trauma, family history of TGCT, and family history of cryptorchidism. We used the same adjusted model for all analyses.

**Table 1:** Characteristics of TGCT cases and controls (group A and group B), TESTIS study, N=1124

Characteristics	TGCT cases (n=454)	Group A controls (n=384)	Group B controls (n=286)	P value cases/all controls*	P value controls A/controls B*
	n (%)	n (%)	n (%)		
Age at diagnosis (cas)/inclusion (controls) (years)				**<0.001**	0.69
≤25	64 (14.1)	20 (5.2)	12 (4.2)		
26–30	106 (23.4)	80 (20.8)	64 (22.4)		
31–35	113 (24.9)	131 (34.1)	108 (37.8)		
36–40	85 (18.7)	95 (24.7)	67 (23.4)		
≥41	43 (9.5)	58 (15.1)	35 (12.2)		
Missing	43 (9.5)	0 (0.0)	0 (0.0)		
Year of birth				**<0.001**	0.15
<1975	37 (8.2)	33 (8.6)	17 (5.9)		
1975–1979	80 (17.6)	82 (21.4)	46 (16.1)		
1980–1984	117 (25.8)	119 (31.0)	99 (34.6)		
1985–1989	130 (28.6)	105 (27.3)	98 (34.3)		
1990–1994	70 (15.4)	40 (10.4)	23 (8.0)		
1995–1999	20 (4.4)	5 (1.3)	3 (1.1)		
Education				0.10	**0.002**
Secondary	180 (39.7)	137 (35.7)	66 (23.1)		
1–2 years university degree	97 (21.4)	91 (23.7)	57 (19.9)		
> 3 years university degree	128 (28.2)	113 (29.4)	121 (42.3)		
Other	48 (10.6)	43 (11.2)	42 (14.7)		
Missing	1 (0.2)	0 (0.0)	0 (0.0)		
Smoking status				0.12	0.10
Former smoker	102 (22.5)	99 (25.8)	72 (25.2)		
Current smoker	147 (32.4)	116 (30.2)	69 (24.1)		
Never smoker	205 (45.2)	169 (44)	145 (50.7)		
Birth weight (g)				0.49	0.99
<2500	25 (5.5)	20 (5.2)	13 (4.6)		
2500–4000	356 (78.4)	318 (82.8)	243 (85)		
≥4000	46 (10.1)	32 (8.3)	26 (9.1)		
Missing	27 (6.0)	14 (3.7)	4 (1.4)		
Gestational age (weeks)				0.16	0.25
≤36	32 (7.1)	23 (6.0)	11 (3.9)		
>36	415 (91.4)	358 (93.2)	271 (94.8)		
Missing	7 (1.5)	3 (0.8)	4 (1.4)		
Born from multiple pregnancy				**0.02**	0.59
No	433 (95.4)	375 (97.7)	277 (96.9)		
Yes	21 (4.6)	8 (2.1)	9 (3.2)		
Missing	0 (0.0)	1 (0.3)	0 (0.0)		
Birth order				**0.04**	0.39
First	213 (46.9)	168 (43.8)	137 (47.9)		
Second	163 (35.9)	135 (35.2)	89 (31.1)		
Third	63 (13.9)	51 (13.3)	42 (14.7)		
Fourth and more	15 (3.3)	30 (7.8)	18 (6.3)		
Sibship size				**0.03**	0.70
1	25 (5.5)	36 (9.4)	23 (8.0)		
2	192 (42.3)	139 (36.2)	115 (40.2)		
3	153 (33.7)	116 (30.2)	90 (31.5)		
≥4	84 (18.5)	92 (24)	58 (20.3)		
Missing	0 (0.0)	1 (0.3)	0 (0.0)		
Personal history of inguinal hernia				**0.01**	0.20
No	414 (91.2)	362 (94.3)	275 (96.2)		
Yes	39 (8.6)	22 (5.7)	10 (3.5)		
Missing	1 (0.2)	0 (0.0)	1 (0.4)		
Personnal history of testicular trauma				**0.004**	0.53
No	387 (85.2)	351 (91.4)	260 (90.9)		
Yes	67 (14.8)	33 (8.6)	26 (9.1)		
Family history of TGCT				**<0.001**	0.33
No	419 (92.3)	375 (97.7)	276 (96.5)		
Yes	33 (7.3)	7 (1.8)	9 (3.2)		
Missing	2 (0.4)	2 (0.5)	1 (0.4)		
Family history of cryptorchidism				**0.01**	0.76
No	422 (93)	369 (96.1)	276 (96.5)		
Yes	28 (6.2)	10 (2.6)	8 (2.8)		
Missing	4 (0.9)	5 (1.3)	2 (0.7)		
Age at voice change (years)				0.61	0.97
<12	16 (3.5)	17 (4.4)	9 (3.2)		
12–16	375 (82.6)	301 (78.4)	231 (80.8)		
>16	53 (11.7)	56 (14.6)	38 (13.3)		
Missing	10 (2.2)	10 (2.6)	8 (2.8)		
Cannabis use at adolescence (12–17 years)				0.30	0.30
No	312 (68.7)	251 (65.4)	192 (67.1)		
Yes	142 (31.3)	133 (34.6)	94 (32.9)		
Cannabis use (18–25 years)				0.68	0.33
No	262 (57.71)	219 (57.03)	174 (60.84)		
Yes	192 (42.29)	164 (42.71)	112 (39.16)		
Missing	0 (0.0)	1 (0.26)	0 (0.0)		
Alcohol consumption				**0.03**	0.15
No	17 (1.52)	23 (3.43)	24 (3.58)		
Yes	429 (38.44)	361 (53.88)	262 (39.10)		

Boldface indicates p-value <0.05.

*P values from univariate conditional logistic regression models—except for age at diagnosis/inclusion, age at inclusion and year of birth which were matching factors.

TGCT, testicular germ cell tumour.

### Statistical analysis

Descriptive statistics (mean and SD for continuous and frequency and percentage for categorical variables) summarising participants’ characteristics were used. In the individual matching, some cases could not be matched to controls and vice versa. Therefore, we performed instead frequency matching on birth year grouped into 5-year categories and region of recruitment. Conditional logistic regression models were used to estimate the OR and 95% CI for ever being employed in a specific occupation/industry compared with not (reference). Each of the jobs reported in the work history was considered. To obtain comparable exposure periods for cases and controls within 5-year age strata, we only considered exposures occurring before the index date (ie, date of TGCT diagnosis for cases, considering; the median age of cases at diagnosis in each stratum for controls).

As the subjects included in the TESTIS study are young, the duration of employment is short. Thus, duration of employment was assessed as an ordinal variable by using the median as cut point (never (reference), <2 years, ≥2 years) for each digit of both codes. Tests for trend were performed by applying the Wald χ^2^ test to the duration variable as an ordinal variable.

Given the broad age strata, we further adjusted for age at index date to avoid residual confounding by age (online supplemental table S1). Adjusted analyses were limited to subjects with no missing data for the adjustment variables and complete occupational history (N=12 and 33 exclusions, respectively). Analyses were repeated for TGCT histological subtype. Associations heterogeneity was tested using polytomous logistic regression for matched case–control studies (SAS macro %subtype).^27^ P values for heterogeneity were derived from the likelihood ratio test.^27^ We additionally conducted sensitivity analyses: excluding cases with a personal history of cryptorchidism (N=40), and TGCT cases not confirmed by pathology reports (N=43).

Data analysis used SAS statistical software V.9.4 (SAS Institute), p values were two sided and considered statistically significant if lower than 0.05.

## Results

### Characteristics of the study population

The mean age (±SD) of cases and group A and group B controls were 31.1 (±6.3), 33.3 (±5.6) and 32.9 (±5.2) years, respectively (table 1). Cases were younger than controls (p<0.001). No difference between the two control groups was observed, except for education level (p=0.002). Although most participants were first born and single born, cases and controls differed for these characteristics (p=0.04 and 0.02, respectively), as well as for sibship size (p=0.03). Cases reported greater alcohol consumption (p=0.03) and more testicular trauma (p=0.004) than controls. Family history of TGCT and of cryptorchidism were more frequent among cases than controls (p<0.001 and 0.01, respectively). Finally, there was no difference between cases and controls in age at voice change, smoking status, cannabis use, birth weight and gestational age.

### TGCT risk by occupation (ISCO68)

There was a positive association between TGCT risk and working as ‘salesmen, shop assistants and demonstrators’ (ISCO: 4–51; OR 1.84; 95% CI (1.20 to 2.82)), particularly for non-seminoma (ISCO: 4–51; OR 2.08; 95% CI (1.16 to 3.75)) (table 2, online supplemental table S1). The subcategory of ‘other salesmen, shop assistants and demonstrators’ (ISCO: 4–51.90) showed the strongest association, both with TGCT overall (OR 2.54; 95% CI (1.38 to 4.65)) and non-seminoma subtype (OR 4.34; 95% CI (2.00 to 9.42)), although CIs were wide. There was a positive association between TGCT (OR 1.71; 95% CI (1.02 to 2.82)), non-seminoma (OR 2.12; 95% CI (1.11 to 4.04)) and ‘agricultural and animal husbandry workers’ (ISCO: 6–2). A positive association was further observed between TGCT risk and ‘production and related workers, transport equipment operators and labourers’ (ISCO: 7/8/9; OR 1.35; 95% CI (1.04 to 1.76)), whereof ‘machinery fitters, machine assemblers’ and ‘electrical fitters, related electrical, electronic workers’ (ISCO: 8–41; OR 2.46; 95% CI (1.08 to 5.62)/ISCO: 8–5; OR 1.80; 95% CI (1.11 to 2.91)). Finally, none of the observed associations showed heterogeneity between histologic subtypes.

**Table 2:** ORs and 95% CIs for TGCT associated with occupations, overall and according to histological subtypes, TESTIS study

Occupation description (ISCO-68)*	All TGCT cases		Seminomas†		Non-seminomas‡		P-HET§
	Ca/Co (%ever)	OR (95% CI)¶	Ca/Co (%ever)	OR (95% CI) ¶	Ca/Co (%ever)	OR (95% CI)¶	
Professional, technical and related workers (0/1)	39.5/45.4	0.83 (0.63 to 1.08)	40.7/45.4	0.82 (0.59 to 1.15)	36.4/45.4	0.78 (0.53 to 1.14)	0.82
Architects, engineers and related technicians (0–2/0–3)	12.4/12.8	0.94 (0.63 to 1.40)	13.6/12.8	1.16 (0.72 to 1.89)	9.1/12.8	0.64 (0.34 to 1.21)	0.14
Doctors, dentists, veterinarians and similar workers (0–6/0–7)	4.0/6.9	0.58 (0.32 to 1.05)	3.3/6.9	0.48 (0.21 to 1.12)	6.1/6.9	0.83 (0.39 to 1.76)	0.34
Teachers (1–3)	6.9/8.8	0.83 (0.51 to 1.35)	6.5/8.8	0.65 (0.34 to 1.26)	6.7/8.8	0.89 (0.44 to 1.80)	0.53
Secondary education teachers (1–32)	4.5/4.3	1.09 (0.57 to 2.05)	4.7/4.3	0.89 (0.39 to 2.04)	4.2/4.3	1.29 (0.53 to 3.14)	0.56
Administrative and managerial workers (2)	12.9/16.3	0.86 (0.59 to 1.26)	15.0/16.3	0.97 (0.61 to 1.54)	9.7/16.3	0.75 (0.41 to 1.37)	0.51
Clerical and related workers (3)	20.5/19.5	1.02 (0.74 to 1.40)	21.5/19.5	0.99 (0.66 to 1.48)	18.8/19.5	0.98 (0.62 to 1.57)	0.99
Sales workers (4)	21.0/17.2	1.28 (0.92 to 1.77)	22.0/17.2	1.12 (0.74 to 1.67)	20/17.2	1.39 (0.87 to 2.20)	0.49
Salesmen, shop assistants and related workers (4–5)	13.1/8.2	**1.66 (1.09 to 2.52)**	12.6/8.2	1.29 (0.77 to 2.15)	12.7/8.2	**1.91 (1.07 to 3.42)**	0.32
Salesmen, shop assistants and demonstrators (4–51)	13.1/7.2	**1.84 (1.20 to 2.82)**	12.6/7.2	1.44 (0.86 to 2.43)	12.7/7.2	**2.08 (1.16 to 3.75)**	0.36
Other salesmen, shop assistants and demonstrators (4–51.90)	7.6/2.9	**2.54 (1.38 to 4.65)**	6.5/2.9	1.71 (0.82 to 3.57)	9.1/2.9	**4.34 (2.00 to 9.42)**	0.09
Service workers (5)	24.0/21.5	1.17 (0.86 to 1.58)	22.9/21.5	0.94 (0.64 to 1.39)	27.9/21.5	1.43 (0.94 to 2.16)	0.15
Cooks, waiters, bartenders and related workers (5–3)	10.2/8.9	1.21 (0.78 to 1.86)	9.3/8.9	0.95 (0.54 to 1.67)	13.3/8.9	1.50 (0.86 to 2.63)	0.26
Waiters, bartenders and related workers (5–32)	6.4/4.5	1.65 (0.94 to 2.90)	6.5/4.5	1.37 (0.69 to 2.74)	7.9/4.5	1.72 (0.83 to 3.57)	0.65
Waiter, general (5–32.10)	5.0/2.9	1.93 (0.99 to 3.74)	3.7/2.9	1.20 (0.49 to 2.90)	7.9/2.9	**2.80 (1.29 to 6.09)**	0.15
Protective service workers (5–8)	8.3/7.4	1.10 (0.69 to 1.77)	8.9/7.4	1.04 (0.58 to 1.87)	7.3/7.4	1.11 (0.55 to 2.24)	0.90
Firefighters (5–81)	3.1/1.7	1.59 (0.69 to 3.69)					
Agricultural, animal husbandry and forestry workers (6)	10.7/6.0	**1.71 (1.07 to 2.72)**	10.3/6.0	1.51 (0.85 to 2.68)	12.7/6.0	**1.94 (1.05 to 3.61)**	0.55
Farmers (6–1)	2.4/1.4	1.84 (0.71 to 4.76)					
Agricultural and animal husbandry workers (6–2)	8.6/4.8	**1.71 (1.02 to 2.86)**	6.5/4.8	1.25 (0.63 to 2.51)	12.1/4.8	**2.12 (1.11 to 4.04)**	0.27
Production and related workers, transport equipment operators and labourers (7/8/9)	48.6/40.8	**1.35 (1.04 to 1.76)**	46.3/40.8	1.23 (0.88 to 1.72)	51.5/40.8	1.43 (0.99 to 2.07)	0.56
Production supervisors and general foremen (7–0)	5.0/4.6	1.24 (0.69 to 2.25)	4.7/4.6	1.12 (0.52 to 2.43)	6.1/4.6	1.29 (0.59 to 2.83)	0.81
Machinery fitters, machine assemblers and precision-instrument makers (except electrical) (8–4)	13.8/8.8	**1.72 (1.14 to 2.59)**	13.6/8.8	**1.74 (1.05 to 2.88)**	13.3/8.8	1.41 (0.80 to 2.48)	0.59
Machinery fitters and machine assemblers (8–41)	3.6/1.7	**2.46 (1.08 to 5.62)**	2.3/1.7	1.81 (0.58 to 5.64)	3.0/1.7	1.58 (0.49 to 5.08)	0.87
Refrigeration and air-conditioning plan installer and mechanic (8–41.80)	2.6/1.2	2.43 (0.92 to 6.39)					
Motor-vehicle mechanics (8–43)	3.3/2.9	1.16 (0.56 to 2.39)					
Machinery fitters, machine assemblers and precision-instrument makers (except electrical) N.E.C. (8–49)	7.6/4.9	1.60 (0.94 to 2.73)	7.5/4.9	1.58 (0.82 to 3.06)	8.5/4.9	1.64 (0.80 to 3.37)	0.94
Electrical fitters and related electrical and electronics workers (8–5)	10.0/5.7	**1.80 (1.11 to 2.91)**	9.3/5.7	1.65 (0.90 to 3.00)	10.3/5.7	1.71 (0.90 to 3.25)	0.94
Plumbers, welders, sheet-netal and structural metal preparers and erectors (8–7)	7.6/4.6	1.67 (0.97 to 2.87)	7.0/4.6	1.29 (0.65 to 2.55)	7.2/4.6	1.82 (0.86 to 3.83)	0.50
Welders and flame-cutters (8–72)	2.9/1.8	1.40 (0.59 to 3.29)	2.8/1.8	1.16 (0.40 to 3.36)	3.0/1.8	2.41 (0.74 to 7.81)	0.37
Sheet-metal workers (8–73)	1.4/1.2	1.31 (0.43 to 4.00)					
Painters (9–3)	1.9/2.2	0.70 (0.28 to 1.74)					
Bricklayers, carpenters and other construction workers (9-5)	9.5/7.5	1.27 (0.80 to 2.01)	8.4/7.5	1.19 (0.65 to 2.17)	10.9/7.5	1.38 (0.74 to 2.59)	0.74
Material handling and related equipment operators, dockers and freight handlers (9–7)	11.0/8.6	1.28 (0.83 to 1.97)	9.3/8.6	1.01 (0.57 to 1.79)	12.1/8.6	1.50 (0.83 to 2.69)	0.35
Transport equipment operators (9–8)	8.1/7.8	1.03 (0.64 to 1.65)	8.9/7.8	0.96 (0.53 to 1.73)	9.1/7.8	1.38 (0.73 to 2.62)	0.42
Laboureres N.E.C (9–9)	2.9/2.5	1.07 (0.48 to 2.38)					

Boldface indicates p-value <0.05.

*Total number of subjects presented for each code can vary due to the management of codes with missing digits. Subjects with a single job coded as missing data, were excluded from the analyses (N=33).

†219 cases of seminoma TGCT were present in the TESTIS study.

‡191 cases of non-seminoma TGCT were present in the TESTIS study.

§P value for heterogeneity derived from the likelihood ratio test, comparing seminoma versus non-seminoma tumours.

¶Estimates obtained comparing TGCT cases to group A and group B controls combined and adjusted for sibship size, being born from multiple pregnancy, personal history of testicular trauma, family history of TGCT and family history of cryptorchidism. Analysis was restricted to subjects with no missing data for the adjustment variables (N=12). Results presented if a job was held by more than five cases and five controls (grey line if less).

Ca/Co (%ever), percentage of cases/controls ever employed; ISCO, International Standard Classification of Occupations; N.E.C, not elsewhere classified; P-HET, P-value for heterogeneity; TGCT, testicular germ cell tumour.

Analyses by duration of employment (table 3) showed a positive association between TGCT and subjects who had been employed for ≥2 years as ‘production and related workers, transport equipment operators and labourers’ (ISCO: 7/8/9; OR_≥ 2 years_=1.45; 95% CI (1.09 to 1.94); p for trend=0.03), whereof ‘electrical fitters and related electrical and electronics workers’, however, with a non-significant duration trend (ISCO: 8–5; OR_≥2 years_=1.83; 95% CI (1.01 to 3.32); p for trend=0.06). A positive association between TGCT risk and duration of employment of less than 2 years for: ‘stock clerks’ (ISCO: 3–91; OR_<2 years_=2.27; 95% CI (1.14 to 4.50); p*-*for-trend=0.03); ‘other salesmen, shop assistants and demonstrators’ (ISCO: 4–51.90; OR_< 2 years_=3.58; CI (1.59 to 8.06); p for trend <0.01) and for ‘machinery fitters, machine assemblers and precision-instrument makers’ (ISCO: 8–4; OR_<2 years_=2.50; 95% CI (1.24 to 5.06); p for trend=0.02) was observed. However, the CIs were wide, indicating substantial uncertainty around the risk estimates.

**Table 3:** ORs and 95% CIs for TGCT associated with occupations, according to employment duration (<2 vs ≥2 years), TESTIS study

Occupation description (ISCO-68)*	Ca/Co (never)	Ca/Co<2 Years	OR1 (95% CI)†	Ca/Co≥2 Years	OR2 (95% CI)†	P trend
Professional, technical and related workers (0/1)	254/355	27/42	0.81 (0.48 to 1.39)	139/253	0.83 (0.62 to 1.10)	0.38
Architects, engineers and related technicians (0–2/0–3)	368/567	12/22	0.72 (0.33 to 1.50)	40/61	1.03 (0.66 to 1.62)	0.68
Teachers (1–3)	391/593	7/20	0.62 (0.25 to 1.50)	22/37	0.94 (0.53 to 1.68)	0.56
Administrative and managerial workers (2)	366/544	13/26	0.82 (0.41 to 1.68)	41/80	0.88 (0.57 to 1.34)	0.74
Clerical and related workers (3)	334/523	38/42	1.37 (0.85 to 2.22)	48/85	0.84 (0.87 to 1.26)	0.27
Sales workers (4)	332/538	30/37	1.28 (0.76 to 2.15)	58/75	1.28 (0.87 to 1.88)	0.34
Salesmen, shop assistants and related workers (4–5)	365/597	25/22	1.81 (0.98 to 3.33)	30/31	1.54 (0.90 to 2.66)	0.06
Salesmen, shop assistants and demonstrators (4–51)	365/603	25/18	**2.12 (1.12 to 4.02)**	30/29	1.65 (0.95 to 2.87)	**0.02**
Retail trade salesman (4–51.30)	391/622	16/13	1.77 (0.82 to 3.81)	13/15	1.33 (0.61 to 2.92)	0.27
Other salesmen, shop assistants and demonstrators (4–51.90)	388/631	22/9	**3.58 (1.59 to 8.06)**	10/10	1.55 (0.61 to 3.95)	**<0.01**
Service workers (5)	319/510	32/37	1.31 (0.78 to 2.20)	69/103	1.11 (0.78 to 1.58)	0.53
Cooks, waiters, bartenders and related workers (5–3)	377/592	18/21	1.28 (0.66 to 2.51)	25/37	1.16 (0.67 to 2.00)	0.68
Waiters, bartenders and related workers (5–32)	393/621	13/9	**2.58 (1.07 to 6.24)**	14/20	1.22 (0.58 to 2.53)	0.10
Waiter, general (5–32.10)	399/631	13/7	**3.38 (1.28 to 8.94)**	8/12	1.11 (0.43 to 2.86)	0.05
Protective service workers (5–8)	385/602	7/10	1.11 (0.40 to 3.10)	28/38	1.10 (0.65 to 1.87)	0.92
Protective service workers N.E.C (5–89)	396/617	8/10	1.41 (0.52 to 3.79)	16/23	1.04 (0.52 to 2.04)	0.79
Watchman (5–89.40)	409/634	6/6	1.25 (0.37 to 4.22)	5/10	1.00 (0.34 to 2.99)	0.94
Agricultural, animal husbandry and forestry workers (6)	375/611	13/11	1.71 (0.73 to 3.98)	32/28	1.71 (0.99 to 2.95)	0.08
Agricultural and animal husbandry workers (6–2)	384/619	14/11	1.81 (0.78 to 4.19)	22/20	1.65 (0.86 to 3.15)	0.13
Production and related workers, transport equipment operators and labourers (7/8/9)	216/385	43/66	1.06 (0.68 to 1.66)	161/199	**1.45 (1.09 to 1.94)**	**0.03**
Production supervisors and general foremen (7–0)	399/620	6/13	0.78 (0.29 to 2.13)	15/17	1.62 (0.78 to 3.36)	0.38
Machinery fitters, machine assemblers and precision-instrument makers (except electrical) (8–4)	362/593	23/14	**2.50 (1.24 to 5.06)**	35/43	1.43 (0.87 to 2.34)	**0.02**
Motor-vehicle mechanics (8–43)	406/631	7/9	1.12 (0.39 to 3.17)	7/10	1.19 (0.44 to 3.26)	0.92
Machinery fitters, machine assemblers and precision-instrument makers (except electrical) N.E.C (8–49)	388/618	15/9	**2.65 (1.12 to 6.26)**	17/23	1.16 (0.59 to 2.30)	0.08
Electrical fitters and related electrical and electronics workers (8–5)	378/613	15/14	1.75 (0.81 to 3.76)	27/23	**1.83 (1.01 to 3.32)**	0.06
Electrical wiremen (8–55)	394/626	8/8	1.39 (0.50 to 3.88)	18/16	1.77 (0.87 to 3.60)	0.24
Plumbers, welders, sheet-metal and structural metal preparers and erectors (8–7)	388/620	16/10	2.23 (0.96 to 5.20)	16/20	1.36 (0.67 to 2.75)	0.13
Welders and flame-cutters (8–72)	408/638	7/6	1.31 (0.41 to 4.17)	5/6	1.51 (0.44 to 5.21)	0.73
Bricklayers, carpenters and other construction workers (9–5)	380/601	16/16	1.75 (0.84 to 3.67)	24/33	1.05 (0.60 to 1.86)	0.33
Material handling and related equipment operators, dockers and freight handlers (9–7)	374/594	26/31	1.39 (0.79 to 2.45)	20/25	1.15 (0.61 to 2.18)	0.49
Transport equipment operators (9–8)	386/599	16/29	0.81 (0.42 to 1.59)	18/22	1.31 (0.67 to 2.54)	0.60

Boldface indicates a p value <0.05.

*Total number of subjects presented for each code can vary due to the management of codes with missing digits. Subjects with a single job coded as missing data, were excluded from the analyses (N=33).

†Estimates obtained comparing TGCT cases to group A and group B controls combined and adjusted for sibship size, being born from multiple pregnancy, personal history of testicular trauma, family history of TGCT and family history of cryptorchidism. Analysis was restricted to subjects with no missing data for the adjustment variables (N=12). Results presented if a job was held by more than five cases and five controls.

Ca/Co (ever)/(never), cases/controls ever/never employed; ISCO, International Standard Classification of Occupations; N.E.C, not elsewhere classified; TGCT, testicular germ cell tumour.

### TGCT risk by industries (NAF99)

This analysis suggested an association between TGCT, non-seminoma and ‘agriculture, hunting and related service activities’ (NAF: 01) (table 4, online supplemental table S2). However, CIs were wide and compatible with inverse associations. Although CIs were also wide, ORs suggested associations between TGCT risk and ‘electrical installation work’, ‘installation of heating and air conditioning equipment’ (NAF: 45.3A; OR 1.98; 95% CI (1.12 to 3.50)/NAF: 45.3F; OR 2.95; 95% CI (1.18 to 7.39)). There was a positive association between TGCT and ‘trade, repair of motor vehicles and household goods’ (NAF: 50–52; OR 1.42; 95% CI (1.06 to 1.90)), ‘supermarkets’ (NAF: 52.1D; OR 2.28; 95% CI (1.28 to 4.06)), industries and ‘technical or professional secondary education’ (NAF: 80.2C; OR 1.55; 95% CI (1.03 to 2.34)). The analysis also suggested a positive association with ‘food retailing in specialty stores’ (NAF: 52.2; OR 2.89; 95% CI (1.03 to 8.14)) but the CI was wide, reflecting the statistical uncertainty of this risk estimate. The category of ‘other community, social and personal services’ (NAF: 90–93) was associated with non-seminoma (OR 2.26; 95% CI (1.32 to 3.87)). An inverse association was observed for non-seminoma for ‘other business activities’ (NAF: 74; OR 0.50; 95% CI (0.27 to 0.92)) with heterogeneity in this association (p for heterogeneity=0.04). ‘Hospital activities’ (NAF: 85.1A) were also inversely associated with TGCT (OR 0.56; 95% CI (0.32 to 0.98)) and seminoma (OR 0.33; 95% CI (0.13 to 0.81)); the latter was found different from the association with non-seminoma (p for heterogeneity=0.04).

**Table 4:** ORs and 95% CIs for TGCT associated with industries, overall and cording to histological subtypes, TESTIS study

Industry description (NAF-99 code)*	All TGCT cases		Seminomas‡		Non-seminomas§		P-HET¶
	Ca/Co (%ever)	OR (95% CI)†	Ca/Co (%ever)	OR (95% CI)†	Ca/Co (%ever)	OR (95% CI)†	
Agriculture, hunting and forestry (01, 02)	7.9/5.2	1.43 (0.85 to 2.41)	7.5/5.3	1.32 (0.69 to 2.54)	9.8/5.3	1.64 (0.83 to 3.25)	0.65
Agriculture, hunting and related service activities (01)	7.6/4.8	1.56 (0.91 to 2.67)	7.5/4.8	1.48 (0.76 to 2.88)	9.1/4.8	1.79 (0.88 to 3.64)	0.70
Manufacturing (15–37)	31.3/29.4	1.10 (0.83 to 1.46)	31.3/29.4	1.13 (0.79 to 1.61)	29.3/29.4	1.00 (0.67 to 1.50)	0.66
Publishing, printing and reproduction of recorded media (22)	1.7/1.1	1.67 (0.56 to 4.99)					
Metalworking (28)	3.8/4.6	0.84 (0.44 to 1.61)	3.7/4.7	0.82 (0.35 to 1.89)	4.3/4.7	0.92 (0.37 to 2.34)	0.85
Electricity, gas and water supply (40, 41)	3.6/2.9	1.12 (0.53 to 2.36)	2.3/2.9	0.80 (0.28 to 2.28)	3.7/2.9	0.88 (0.32 to 2.40)	0.90
Electricity, gas, steam and hot water supply (40)	2.9/2.6	0.98 (0.44 to 2.19)	2.3/2.6	0.85 (0.29 to 2.46)	3.0/2.6	0.75 (0.25 to 2.25)	0.88
Construction (45)	20.5/16.6	1.27 (0.91 to 1.77)	16.8/16.4	0.94 (0.61 to 1.47)	22.6/16.4	1.41 (0.89 to 2.22)	0.22
Site preparation (45.1)	1.2/1.2	0.76 (0.23 to 2.50)					
Construction of building and civil engineering works (45.2)	6.2/4.7	1.38 (0.78 to 2.43)	3.3/4.7	0.61 (0.26 to 1.47)	9.1/4.7	1.80 (0.88 to 3.66)	0.06
Installation work (45.3)	11.2/6.4	**1.99 (1.25 to 3.15**)	9.4/6.4	1.52 (0.84 to 2.75)	9.8/6.4	1.59 (0.83 to 3.03)	0.92
Electrical installation work (45.3A)	6.9/4.04	**1.98 (1.12 to 3.50**)	6.6/4.1	1.66 (0.82 to 3.38)	6.1/4.1	1.96 (0.89 to 4.34)	0.76
Installation of heating and air conditioning equipment (45.3F)	3.3/1.2	**2.95 (1.18 to 7.39**)					
Finishing work (45.4)	5.0/5.3	0.87 (0.48 to 1.56)	4.7/5.1	0.82 (0.38 to 1.78)	6.1/5.1	1.15 (0.53 to 2.51)	0.54
Trade, repair of motor vehicles and household goods (50–52)	30.1/23.7	**1.42 (1.06 to 1.90**)	31.8/23.8	1.34 (0.94 to 1.92)	28.0/23.8	1.41 (0.93 to 2.14)	0.86
Motor trade and repair (50)	5.7/4.2	1.40 (0.78 to 2.52)	7.5/4.2	1.59 (0.81 to 3.12)	4.3/4.2	1.16 (0.47 to 2.86)	0.57
Wholesale trade and commercial intermediaries (51)	10.5/8.2	1.35 (0.87 to 2.10)	10.7/8.2	1.25 (0.73 to 2.16)	11.0/8.2	1.44 (0.79 to 2.65)	0.73
Retail trade and repair of household goods (52)	17.7/13.7	1.38 (0.97 to 1.96)	17.3/13.0	1.18 (0.76 to 1.82)	17.1/13.0	1.46 (0.90 to 2.39)	0.52
Retail sale in non-specialised shops (52.1)	7.4/4.3	**1.91 (1.11 to 3.28**)	5.6/4.3	1.29 (0.63 to 2.66)	9.8/4.3	**3.00 (1.51 to 5.98**)	0.10
Supermarkets (52.1D)	7.2/3.5	**2.28 (1.28 to 4.06**)	5.6/3.6	1.64 (0.78 to 3.46)	9.1/3.6	**3.20 (1.55 to 6.60**)	0.21
Food retail in specialised shops (52.2)	2.4/1.1	**2.89 (1.03 to 8.14**)					
Hotels and restaurants (55)	12.2/10.6	1.23 (0.82 to 1.84)	11.2/10.5	1.07 (0.63 to 1.79)	14.0/10.5	1.34 (0.79 to 2.29)	0.56
Transport and communications (60–64)	10.7/14.5	0.74 (0.50 to 1.09)	14.0/14.6	0.94 (0.59 to 1.50)	7.3/14.6	**0.49 (0.26 to 0.94**)	0.10
Financial activities (65–67)	4.8/5.4	1.05 (0.58 to 1.88)	4.7/5.4	0.95 (0.45 to 2.03)	6.1/5.4	1.72 (0.80 to 3.69)	0.29
Real estate, rental and business services (70–74)	24.1/30.2	0.77 (0.57 to 1.03)	29.0/30.3	0.91 (0.63 to 1.31)	17.1/30.3	**0.51 (0.32 to 0.81**)	0.05
Other business activities (74)	14.8/16.6	0.91 (0.64 to 1.30)	17.8/16.7	1.06 (0.69 to 1.63)	8.5/16.7	**0.50 (0.27 to 0.92**)	**0.04**
Public administration (75)	16.9/16.5	0.98 (0.69 to 1.38)	15.9/16.6	0.83 (0.52 to 1.30)	18.9/16.6	1.15 (0.71 to 1.86)	0.33
Education (80)	22.7/23.9	0.97 (0.72 to 1.31)	21.0/24.0	0.78 (0.52 to 1.15)	23.2/24.0	0.99 (0.65 to 1.51)	0.41
Secondary education (80.2)	15.8/12.3	1.33 (0.92 to 1.92)	15.0/12.4	1.03 (0.64 to 1.66)	15.2/12.4	1.27 (0.76 to 2.13)	0.55
General secondary education (80.2A)	3.1/3.3	0.88 (0.41 to 1.88)	2.8/3.3	0.50 (0.18 to 1.41)	3.0/3.3	1.33 (0.46 to 3.86)	0.20
Technical or professional secondary education (80.2C)	12.9/8.9	**1.55 (1.03 to 2.34**)	12.1/8.9	1.31 (0.78 to 2.21)	12.8/8.9	1.35 (0.77 to 2.39)	0.94
Health and social work (85)	7.4/12.9	**0.57 (0.37 to 0.89**)	8.4/13.0	0.64 (0.36 to 1.11)	7.9/13.0	0.61 (0.32 to 1.16)	0.92
Human health activities (85.1)	5.5/9.7	**0.56 (0.33 to 0.93**)	4.7/9.8	**0.45 (0.22 to 0.93**)	7.9/9.8	0.79 (0.41 to 1.52)	0.26
Hospital activities (85.1A)	4.5/7.9	**0.56 (0.32 to 0.98**)	2.8/7.9	0.33 (0.13 to 0.81)	7.9/7.9	1.01 (0.52 to 1.96)	**0.04**
Other community, social and personal services (90–93)	14.1/10.9	**1.48 (1.00 to 2.19**)	13.6/11.0	1.23 (0.75 to 2.01)	16.5/11.0	**2.26 (1.32 to 3.87**)	0.10
Recreational, cultural and sporting activities (92)	10.5/8.6	1.40 (0.91 to 2.17)	9.8/8.7	1.09 (0.62 to 1.90)	12.2/8.7	**2.07 (1.14 to 3.76**)	0.12

Boldface indicates a p value <0.05.

*Total number of subjects presented for each code can vary due to the management of codes with missing digits. Subjects with a single job coded as missing data, were excluded from the analyses (N=33).

†Estimates obtained comparing TGCT cases to group A and group B controls combined and adjusted for sibship size, being born from multiple pregnancy, personal history of testicular trauma, family history of TGCT and family history of cryptorchidism. Analysis was restricted to subjects with no missing data for the adjustment variables (N=12). Results presented if a job was held by more than five cases and five controls (grey line if less).

‡219 cases of seminoma TGCT were present in the TESTIS study.

§191 cases of non-seminoma TGCT were present in the TESTIS study.

¶ P-value for heterogeneity derived from the Likelihood Ratio Test, comparing seminoma versus non-seminoma tumours.

Ca/Co (%ever), percentage of cases/controls ever employed; ISCO, International Standard Classification of Occupations; NAF-99, Nomenclature d’Activités Française-1999; N.E.C, not elsewhere classified; P-HET, P-value for heterogeneity; TGCT, testicular germ cell tumour.

Analyses by duration of employment (online supplemental table S3) showed an association between TGCT risk and subjects who had been employed 2 years or more in ‘installation work’ sector activities (NAF: 45.3; OR_≥2 years_=2.01; 95% CI (1.14 to 3.56); p for trend 0.02). Positive association between TGCT and employment less than 2 years was observed for ‘supermarket’ (NAF: 52.1D; OR_< 2 years_=2.97; 95% CI (1.33 to 6.63); p for trend=0.01). However, the CIs observed were also wide.

### Sensitivity analyses

Most of the previously identified occupational groups/sectors were also identified when cases with a personal history of cryptorchidism were excluded (N=40) (online supplemental table S4 and S5), when cases not confirmed by pathology reports were excluded (N=43) (online supplemental table S6 and S7) or when an adjustment for age at index date was made (online supplemental table S8 and S9). However, a numerically positive association with ‘production and related workers, transport equipment operators and labourers’ (ISCO: 7/8/9) was observed when analyses were restricted to confirmed cases.

## Discussion

Men ever employed in agricultural, production, transport and labourer occupations had an elevated TGCT risk compared with those who never worked there, and it appears to increase with duration of employment. An important strength of our study was to use two distinct classifications: one international (ISCO) for occupation and one French national (NAF) for industry that yielded consistent positive associations for several occupations/industries, even if CIs were sometimes wider in one of the two. Thus, men employed in agricultural occupations showed an increased TGCT risk in both classifications, but with wider CI in NAF. Both classifications further found a positive association between electrical workers and TGCT. The positive association observed for salesmen was also supported by the association observed with the ‘retail in non-specialty stores’ and ‘supermarket’ sectors for TGCT and non-seminoma risk. Finally, both classifications suggested an inverse association in relation to medical/paramedical workers, with wider CI regarding associations found with ISCO.

The observed increased TGCT risk for agricultural workers is consistent with previous studies.^7 28–31^ Conversely, two meta-analyses^32 33^ and a literature review^34^ of cancers among farmers found no evidence of an increased risk, which does not exclude the possibility that exposure to certain agricultural chemicals may be associated with increased TGCT risk.^2 7^ Several pesticides have been classified as EDCs due to their estrogenic or anti-androgenic effects.^35^ Exposure to EDCs has been hypothesised to increase TGCT risk by interfering with the regular hormonal balance.^35^ For instance, a positive association between dichlorodiphenyldichloroethylene, the most persistent metabolite of dichlorodiphenyltrichloroethane (DDT) and TGCT has been observed previously.^2^ Although DDT is now banned effectively in many countries, some older subjects may have been exposed early in their occupational history. While pesticides are the most commonly studied exposure in relation to TGCT among agricultural workers,^7 12^ they may also be exposed to a variety of other potentially carcinogenic substances, including solvents, metals, organic dusts and diesel exhaust fumes.^36^

An increased TGCT risk in non-agricultural workers such as production and related workers, transportation equipment operators, and labourers has also been observed, especially for those who have held these jobs for 2 years or more. Our results were consistent with previous studies suggesting an increased TGCT risk among workers in the electrical and electronics industry and related sectors,^28 31^ but other did not support this finding.^37^ Some studies, based on job titles, suggested that exposure to solvents may increase TGCT risk.^2 12^ Moreover, an increase in TGCT risk for subjects employed in the trade, motor vehicle repair and household goods industries were observed in our study, consistent with the common use of certain solvents as degreasers in these industries.^38^

In contrast to our findings, an association between testicular cancer and firefighters has been suggested in several studies,^39 40^ potentially associated with some complex chemical compounds present in fire smoke.^39^ For example, firefighters may be more exposed to PFOA than the general population. A recent review meta-analysis suggests an average increase in TGCT risk per 10 ng/mL increase in serum PFOA.^8^ Other occupations have been found linked to TGCT risk in our study, although it may be more difficult to relate them to specific occupational exposures. Conversely to a previous study,^31^ our results suggest an inverse association of TGCT with health and social work. Furthermore, the observed excess TGCT risk in white-collar workers (ie, production supervisor and general foreman) is consistent with previous findings.^7 41^ However, this finding is possibly due to socioeconomic status (SES),^31^ yet the association between SES and TGCT risk remains unclear with divergent evidence.^42^

Finally, the results suggest that despite evolving regulation and increased workplace protection from carcinogen exposure,^14^ agricultural workers, production and transport workers such as electrical and electronic are consistently associated with TGCT over time. Nevertheless, humans are exposed to many chemicals, nearly 1500 are suspected EDCs, and this number will continue to grow.^43^ It is, therefore, necessary to conduct further epidemiological studies, with advanced methods of occupational exposure assessment, on the role of chemicals involved in high-risk occupations and TGCT risk, such as solvents or pesticides.

### Strengths and limitations

This study has several strengths. Because of its national coverage (20 recruiting UH) and the participants’ detailed occupational history including tasks performed, it offers an opportunity to investigate and characterise occupations and industry sectors associated with testicular cancer in young adults in recent years. Detailed information allowed accounting for suspected or known TGCT risk factors.

This study has some limitations. The main one was the difficulty to obtain an unbiased random sample of general population controls given the low response rate in the target population.^19 20^ Another limitation was our inability to determine the controls’ representativeness of the source population from which the cases originated; this limitation has been discussed in detail previously.^17^ While it has been suggested that occupational exposure might not be relevant for TGCT because of the young age of patients and the short duration of occupational exposure, the presence of GCNIS early in life and TCGT in young adulthood supports the hypothesis of combined early and later life exposures in the development of TGCT.^2^ Although this limitation is not specific to our study, the method applied identified occupational groups with higher risk due to collective exposure to agents or chemicals, exposure to these also varies within each job or industry, which is not accounted for in this way.^12^ Grouping potentially highly exposed subjects with unexposed from the same job could attenuate the strength of the association. Based on a priori hypothesis, we investigated associations between TGCT and occupations/industries. The use of two complementary nomenclatures, based on broad occupation/industry categories and associated subcategories, involved multiple analyses. Yet, as these variables are related, as well as to allow interpretation of results in light of previous literature, correction for multiple testing and the commonly used Bonferroni method were not considered appropriate.^44^ Therefore, positive associations may have occurred by chance and our results should be interpreted with caution. Of note, most of the associations observed were on jobs for which we had plausible hypotheses, and that have been found in previous studies. There is also a risk of bias due to differential participation in case–control studies. This bias is positively associated with the difference in response rates between groups for the characteristic causing differential participation, and inversely associated with the total response rate in controls in previous studies.^45^ However, status of non-participants (N=96 people who were contacted but did not participate in the study, see online supplemental figure S1) was unfortunately not available to compare response rates between cases and controls. The overall response rate in this study is like that of previous studies.^46–48^ Yet, in the clinical setting, it is likely that additional eligible cases and controls were not interested in participating but not reported by the centres, and our response rate may be overestimated. A previous French hospital-based case–control study on TGCT risk factors, with recruitment of cases in CECOS at time of sperm cryopreservation (between 2002 and 2005), reported a response rate of 81% for cases and of 39% for controls recruited among partners of women in the maternity clinic of the same hospital as the CECOS.^49^ Our occupational data were self-reported and could have been subject to recall bias. However, the use of structured documents to prepare the interview and questionnaires, as well as training of interviewers and telephone interviews blinded to case–control status were intended to minimise recall bias. Thus, it is more likely that recall bias was non-differential with respect to recall of lifetime work history and coding by an industrial hygienist blinded to case–control status, leading instead to null bias.^50^ There is also a difference in the regional distribution of the population, with participating controls more often coming from nearby/urban locations. Some types of jobs may be underrepresented for our control population, such as agricultural jobs, leading to a potential differential job classification error and thus biasing the OR.^50^ Results by duration of employment should also be interpreted with caution. The subjects’ young age resulted in a low median duration of employment, which may have reduced the strength of the association in this analysis. The subtypes show different peak incidences by age (ie, the highest incidence rate of non-seminoma is in the 25–29 years age group, while it is in the 35–39 years age group for seminomas^2^), so the results may be biased by analysing the duration for all TGCT, when we observed a stronger effect in one of the subtypes. We could not verify the influence of histological subtype on risk by duration due to an insufficient number of subjects.

## Conclusion

In conclusion, the findings suggest a possible role of some occupational exposures in the development of TGCT. Men employed in agricultural, electrical and electronics occupations had an elevated TGCT risk compared with those who never worked there, and it seems to be higher for workers employed for 2 years or more. The consistency of these findings with previous studies suggests opportunities to reinforce work health and safety measures to reduce occupational exposure to carcinogens. Also, further research targeting specific exposures is warranted to determine the role of agents or chemical currently involved in the identified high-risk occupations and investigate associated TGCT risk.

## Data Availability

Data are available on reasonable request. The data collection process is described in the Method section of this article.
