# Supplementary material for: Testicular germ cell tumour risk by occupation and industry: a French case–control study – TESTIS
Source: Occup Environ Med. 2023 May 25;80(7):407–17. doi: 10.1136/oemed-2022-108601 (PMC10314033; doi:10.1136/oemed-2022-108601)
Supplement: Supplementary data [file oemed-2022-108601supp001.pdf]

Supplementary material

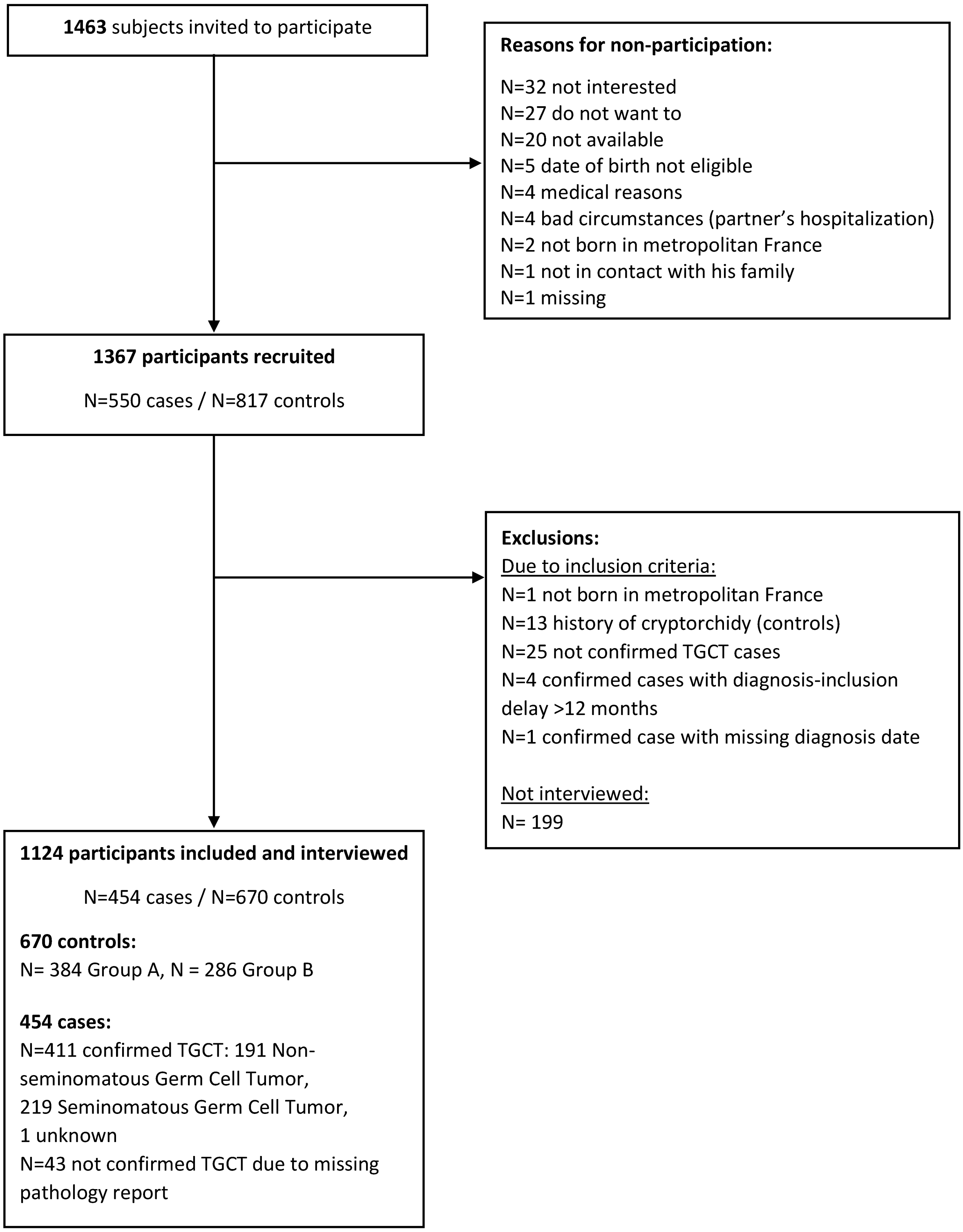

Figure S1. Flow chart of the TESTIS study population

Table S1. Odds ratios and 95% confidence intervals for TGCT associated with occupations, overall and according to histological subtypes, TESTIS study – *full table.*

Occupation description (ISCO-68) [a]	All TGCT cases			Seminomas [c]			Non-seminomas [d]			P-HET[e]
	Ca/Co (never)	*Ca/Co (ever)*	**OR (95% IC) [b]**	*Ca/Co (never)*	*Ca/Co (ever)*	**OR (95% IC) [b]**	*Ca/Co (never)*	*Ca/Co (ever)*	**OR (95% IC) [b]**	
**Professional, Technical and Related Workers (0/1)**	254/355	166/295	0.83 (0.63-1.08)	127/355	87/295	0.82 (0.59-1.15)	105/355	60/295	0.78 (0.53-1.14)	0.82
Architects, engineers and related technicians (0-2/0-3)	368/567	52/83	0.94 (0.63-1.40)	185/567	29/83	1.16 (0.72-1.89)	150/567	15/83	0.64 (0.34-1.21)	0.14
Mechanical engineers (0-24)	412/634	7/16	0.59 (0.22-1.61)							
Draughtsmen (0-32)	408/639	11/11	1.60 (0.65-3.98)	209/639	5/11	1.75 (0.55-5.60)	159/639	5/11	1.97 (0.59-6.59)	0.89
Civil engineering technicians (0-33)	411/637	8/13	0.97 (0.38-2.48)							
Clerk of works (0-33.40)	411/639	8/11	1.05 (0.40-2.76)							
Electrical and electronics engineering technicians (0-34)	413/640	6/10	0.77 (0.26-2.31)							
Engineering technicians N.E.C (0-39)	411/632	8/18	0.73 (0.31-1.74)							
Other engineering technicians (0-39.90)	413/640	6/10	0.92 (0.32-2.65)							
Life scientists and related technicians (0-5)	408/639	12/11	1.40 (0.58-3.41)							
Doctors, dentists, veterinarians and similar workers (0-6/0-7)	403/605	17/45	0.58 (0.32-1.05)	207/605	7/45	0.48 (0.21-1.12)	155/605	10/45	0.83 (0.39-1.76)	0.34
Medical doctors (0-61)	415/634	5/16	0.49 (0.17-1.38)							
Professional nurses (0-71)	414/637	6/13	0.66 (0.24-1.79)							
Professional nurse (general) (0-71.10)	414/639	6/11	0.74 (0.26-2.06)							
Statisticians, mathematicians, systems analysts and related technicians (0-8)	384/597	36/53	1.13 (0.70-1.81)	193/597	21/53	1.40 (0.79-2.49)	152/597	13/53	0.99 (0.50-1.94)	0.43
Systems analysts (0-83)	386/603	34/47	1.23 (0.75-2.01)	194/600	20/47	1.48 (0.82-2.68)	153/600	12/47	1.05 (0.52-2.12)	0.46
Accountants (1-1)	410/640	10/10	1.73 (0.69-4.38)							
Accountants (1-10)	410/640	10/10	1.73 (0.69-4.38)							
Auditor (1-10.20)	410/641	10/9	1.96 (0.76-5.09)							
Teachers (1-3)	391/593	29/57	0.83 (0.51-1.35)	200/593	14/57	0.65 (0.34-1.26)	154/593	11/57	0.89 (0.44-1.80)	0.53
Secondary education teachers (1-32)	401/622	19/28	1.09 (0.57-2.05)	204/622	10/28	0.89 (0.39-2.04)	158/622	7/28	1.29 (0.53-3.14)	0.56

Other secondary education teachers (1-32.90)	408/632	9/15	1.02 (0.42-2.44)							
**Administrative and Managerial Workers (2)**	366/544	54/106	0.86 (0.59-1.26)	182/544	32/106	0.97 (0.61-1.54)	149/544	16/106	0.75 (0.41-1.37)	0.51
Managers (2-1)	366/545	54/105	0.87 (0.60-1.27)	182/545	32/105	0.97 (0.61-1.54)	149/545	16/105	0.76 (0.42-1.39)	0.53
General managers (2-11)	413/622	7/22	0.56 (0.23-1.37)							
Managers N.E.C (2-19)	372/563	48/82	1.03 (0.69-1.55)	187/563	27/82	1.12 (0.68-1.85)	150/563	15/82	0.92 (0.49-1.71)	0.63
Sales manager (except wholesale and retail trade) (2-19.30)	412/636	8/8	1.58 (0.57-4.43)							
Budgeting and accounting manager (2-19.50)	414/629	6/13	0.89 (0.32-2.44)							
Other managers (2-19.90)	385/581	35/61	0.98 (0.62-1.55)	194/581	20/61	1.06 (0.60-1.88)	153/581	12/61	0.96 (0.48-1.91)	0.82
**Clerical and Related Workers (3)**	334/523	86/127	1.02 (0.74-1.40)	168/523	46/127	0.99 (0.66-1.48)	134/523	31/127	0.98 (0.62-1.57)	0.99
Bookkeepers, cashiers and related workers (3-3)	410/629	10/21	0.61 (0.27-1.35)	209/629	5/21	0.47 (0.17-1.31)	160/629	5/21	1.13 (0.39-3.23)	0.25
Bookkeepers and cashiers (3-31)	415/642	5/8	0.70 (0.22-2.28)							
Bookkeepers, cashiers and related workers N.E.C (3-39)	414/637	6/13	0.65 (0.24-1.80)							
Computing machine operators (3-4)	408/634	12/16	1.09 (0.49-2.44)	209/634	5/16	0.70 (0.23-2.11)	160/634	5/16	1.25 (0.42-3.73)	0.46
Clerical and related workers N.E.C (3-9)	355/569	65/81	1.24 (0.86-1.79)	177/569	37/81	1.39 (0.89-2.17)	144/569	21/81	1.00 (0.59-1.72)	0.36
Stock clerks (3-91)	385/609	35/41	1.24 (0.76-2.02)	195/609	19/41	1.39 (0.76-2.53)	154/609	11/41	0.97 (0.47-2.00)	0.45
Dispatching and receiving clerk (3-91.20)	404/627	16/23	1.10 (0.57-2.15)							
Storeroom clerk (3-91.40)	403/632	17/18	1.28 (0.63-2.59)	205/632	9/18	1.32 (0.55-3.19)	159/632	6/18	1.18 (0.45-3.13)	0.87
Correspondence and reporting clerks (3-93)	405/627	15/23	1.08 (0.55-2.13)							
Office clerk (general) (3-93.10)	408/633	12/17	1.12 (0.52-2.42)							
Receptionists and travel agency clerks (3-94)	403/628	17/22	1.14 (0.58-2.24)	205/628	9/22	1.14 (0.49-2.62)	157/628	8/22	1.34 (0.54-3.32)	0.79
Receptionist (general) (3-94.10)	407/640	13/10	1.88 (0.79-4.49)	207/640	7/10	1.71 (0.61-4.75)	159/640	6/10	2.28 (0.72-7.17)	0.71
**Sales Workers (4)**	332/538	88/112	1.28 (0.92-1.77)	167/535	47/112	1.12 (0.74-1.67)	132/535	33/112	1.39 (0.87-2.20)	0.49
Working proprietors (wholesale and retail trade) (4-1)	413/642	7/8	1.71 (0.58-5.02)							
Technical Salesmen, Commercial Travellers and Manufacturers' Agents (4-3)	388/597	32/53	0.93 (0.58-1.50)	196/597	18/53	0.92 (0.51-1.66)	152/597	13/53	0.95 (0.49-1.86)	0.94
Technical Salesmen and Service Advisers (4-31)	388/599	32/51	0.97 (0.60-1.56)	196/599	18/51	0.96 (0.53-1.73)	152/599	13/51	0.98 (0.50-1.90)	0.97
Insurance, Real Estate, Securities and Business Services Salesmen and Auctioneers (4-4)	414/638	6/12	0.80 (0.29-2.22)							

Insurance, Real Estate and Securities Salesmen (4-41)	414/639	6/11	0.86 (0.31-2.42)							
Salesmen, Shop Assistants and Related Workers (4-5)	365/597	55/53	1.66 (1.09-2.52)	187/597	27/53	1.29 (0.77-2.15)	144/597	21/53	1.91 (1.07-3.42)	0.32
Salesmen, Shop Assistants and Demonstrators (4-51)	365/603	55/47	1.84 (1.20-2.82)	187/603	27/47	1.44 (0.86-2.43)	144/603	21/47	2.08 (1.16-3.75)	0.36
Retail trade salesman (4-51.30)	391/622	29/28	1.54 (0.89-2.68)	200/622	14/28	1.32 (0.67-2.61)	156/622	9/28	1.23 (0.55-2.77)	0.90
Other salesmen, shop assistants and demonstrators (4-51.90)	388/631	32/19	2.54 (1.38-4.65)	200/631	14/19	1.71 (0.82-3.57)	150/631	15/19	4.34 (2.00-9.42)	0.09
**Service Workers (5)**	319/510	101/140	1.17 (0.86-1.58)	165/510	49/140	0.94 (0.64-1.39)	119/510	46/140	1.43 (0.94-2.16)	0.15
Cooks, Waiters, Bartenders and Related Workers (5-3)	377/592	43/58	1.21 (0.78-1.86)	194/592	20/58	0.95 (0.54-1.67)	143/592	22/58	1.50 (0.86-2.63)	0.26
Cooks pressers (5-31)	396/616	24/34	1.09 (0.62-1.91)	205/616	9/34	0.69 (0.31-1.53)	151/616	14/34	1.72 (0.86-3.44)	0.09
Cook, except private service (5-31.30)	406/636	14/14	1.63 (0.74-3.56)	208/636	6/14	0.98 (0.35-2.77)	157/636	8/14	2.53 (0.99-6.48)	0.18
Other cooks (5-31.90)	405/624	15/26	0.84 (0.43-1.66)	209/624	5/26	0.50 (0.18-1.41)	156/624	9/26	1.35 (0.60-3.10)	0.13
Waiters, Bartenders and Related Workers Workers (5-32)	393/621	27/29	1.65 (0.94-2.90)	200/621	14/29	1.37 (0.69-2.74)	152/621	13/29	1.72 (0.83-3.57)	0.65
Waiter, general (5-32.10)	399/631	21/19	1.93 (0.99-3.74)	206/631	8/19	1.20 (0.49-2.90)	152/631	13/19	2.80 (1.29-6.09)	0.15
Bartender (5-32.50)	410/640	10/10	2.01 (0.81-5.02)							
Building Caretakers, Charworkers, Cleaners and Related Workers (5-5)	382/594	38/56	1.08 (0.69-1.69)	197/594	17/56	0.79 (0.44-1.43)	145/594	20/56	1.40 (0.79-2.47)	0.17
Charworkers, Cleaners and Related Workers (5-52)	382/594	38/56	1.08 (0.69-1.69)	197/594	17/56	0.79 (0.44-1.43)	145/594	20/56	1.40 (0.79-2.47)	0.17
Charworker (5-52.20)	386/595	34/55	0.98 (0.62-1.55)	198/595	16/55	0.76 (0.41-1.40)	147/595	18/55	1.25 (0.69-2.27)	0.25
Protective Service Workers (5-8)	385/602	35/48	1.10 (0.69-1.77)	195/602	19/48	1.04 (0.58-1.87)	153/602	12/48	1.11 (0.55-2.24)	0.90
Fire-Fighters (5-81)	407/639	13/11	1.59 (0.69-3.69)							
Fire-fighter (general) (5-81.10)	408/639	12/11	1.42 (0.60-3.34)							
Protective Service Workers N.E.C (5-89)	396/617	24/33	1.14 (0.65-2.01)	198/617	16/33	1.32 (0.69-2.53)	157/617	8/33	1.16 (0.50-2.69)	0.81
Watchman (5-89.40)	409/634	11/16	1.10 (0.49-2.49)							
Other protective service workers (5-89.90)	405/635	15/15	1.53 (0.72-3.27)	205/635	9/15	1.62 (0.66-3.95)	159/635	6/15	1.96 (0.69-5.52)	0.79
Service Workers N.E.C (5-9)	412/628	8/22	0.56 (0.24-1.32)							
Other Service Workers (5-99)	412/631	8/19	0.63 (0.27-1.51)							

**Agricultural, Animal Husbandry and Forestry Workers (6)**	375/611	45/39	1.71 (1.07-2.72)	192/611	22/39	1.51 (0.85-2.68)	144/611	21/39	1.94 (1.05-3.61)	0.55
Farmers (6-1)	410/641	10/9	1.84 (0.71-4.76)							
Specialised Farmers (6-12)	414/644	6/6	1.68 (0.52-5.48)							
Agricultural and Animal Husbandry Workers (6-2)	384/619	36/31	1.71 (1.02-2.86)	200/619	14/31	1.25 (0.63-2.51)	145/619	20/31	2.12 (1.11-4.04)	0.27
Nursery Workers and Gardeners (6-27)	401/635	19/15	1.74 (0.85-3.54)	204/635	10/15	1.60 (0.67-3.81)	157/635	8/15	1.89 (0.72-4.95)	0.81
Gardener (6-27.40)	402/636	18/14	1.70 (0.81-3.53)	205/636	9/14	1.54 (0.62-3.78)	157/636	8/14	1.89 (0.72-4.95)	0.76
**Production and related workers, transport equipment operators and labourers (7/8/9)**	216/385	204/265	1.35 (1.04-1.76)	115/385	99/265	1.23 (0.88-1.72)	80/ 385	85/265	1.43 (0.99-2.07)	0.56
Production Supervisors and General Foremen (7-0)	399/620	21/30	1.24 (0.69-2.25)	204/620	10/30	1.12 (0.52-2.43)	155/620	10/30	1.29 (0.59-2.83)	0.81
Production Supervisors and General Foremen (7-00)	399/620	21/30	1.24 (0.69-2.25)	204/620	10/30	1.13 (0.52-2.43)	155/620	10/30	1.29 (0.59-2.83)	0.81
Production supervisor and general foreman (general) (7-00.10)	406/638	14/12	2.32 (1.05-5.15)	206/638	8/12	2.58 (1.00-6.69)	159/638	6/12	2.06 (0.73-5.80)	0.75
Food and Beverage Processers (7-7)	409/627	11/23	0.76 (0.36-1.64)							
Cabinetmakers and Related Woodworkers (8-1)	415/645	5/5	1.45 (0.39-5.44)							
Blacksmiths, Toolmakers and Machine Tool Operators (8-3)	408/633	12/17	0.84 (0.37-1.91)							
Machinery Fitters, Machine Assemblers and Precision-Instrument Makers (except Electrical) (8-4)	362/593	58/57	1.72 (1.14-2.59)	185/593	29/57	1.74 (1.05-2.88)	143/593	22/57	1.41 (0.80-2.48)	0.59
Machinery Fitters and Machine Assemblers (8-41)	405/639	15/11	2.46 (1.08-5.62)	209/639	5/11	1.81 (0.58-5.64)	160/639	5/11	1.58 (0.49-5.08)	0.87
Refrigeration and air-conditioning plan installer and mechanic (8-41.80)	409/642	11/8	2.43 (0.92-6.39)							
Motor-Vehicle Mechanics (8-43)	406/631	14/19	1.16 (0.56-2.39)							
Automobile mechanic (8-43.20)	413/639	7/11	1.03 (0.39-2.76)							
Motor-truck mechanic (8-43.30)	413/644	7/6	1.68 (0.54-5.24)							
Machinery Fitters, Machine Assemblers and Precision-Instrument Makers (except Electrical) N.E.C. (8-49)	388/618	32/32	1.60 (0.94-2.73)	198/618	16/32	1.58 (0.82-3.06)	151/618	14/32	1.64 (0.80-3.37)	0.94
Agricultural machinery mechanic (8-49.55)	415/645	5/5	1.64 (0.46-5.94)							

Plant maintenance mechanic (8-49.70)	405/633	15/17	1.37 (0.65-2.86)	209/633	5/17	0.90 (0.31-2.60)	157/633	8/17	1.54 (0.60-3.92)	0.46
Electrical Fitters and Related Electrical and Electronics Workers (8-5)	378/613	42/37	1.80 (1.11-2.91)	194/613	20/37	1.65 (0.90-3.00)	148/613	17/37	1.71 (0.90-3.25)	0.94
Electrical Wiremén (8-55)	394/626	26/24	1.64 (0.91-2.97)	199/626	15/24	1.87 (0.93-3.75)	157/626	8/24	1.36 (0.56-3.26)	0.57
Building electrician (8-55.20)	400/629	20/21	1.47 (0.77-2.81)	202/629	12/21	1.71 (0.80-3.66)	160/629	5/21	0.96 (0.34-2.68)	0.37
Plumbers, Welders, Sheet-Metal and Structural Metal Preparers and Erectors (8-7)	388/620	32/30	1.67 (0.97-2.87)	199/620	15/30	1.29 (0.65-2.55)	153/620	12/30	1.82 (0.86-3.83)	0.50
Plumbers and Pipe Fitters (8-71)	406/638	14/12	1.70 (0.75-3.84)	208/638	6/12	1.40 (0.49-4.00)	160/638	5/12	1.28 (0.42-3.92)	0.91
Plumber (general) (8-71.05)	406/641	14/9	2.35 (0.98-5.65)	208/641	6/9	2.13 (0.70-6.43)	160/641	5/9	1.65 (0.51-5.27)	0.75
Welders and Flame-Cutters (8-72)	408/638	12/12	1.40 (0.59-3.29)	208/638	6/12	1.16 (0.40-3.36)	160/638	5/12	2.41 (0.74-7.81)	0.37
Sheet-Metal Workers (8-73)	414/642	6/8	1.31 (0.43-4.00)							
Rubber and Plastics Product Makers (9-0)	414/645	6/5	2.09 (0.62-7.11)							
Painters (9-3)	412/636	8/14	0.70 (0.28-1.74)							
Bricklayers, Carpenters and Other Construction Workers (9-5)	380/601	40/49	1.27 (0.80-2.01)	196/601	18/49	1.19 (0.65-2.17)	147/601	18/49	1.38 (0.74-2.59)	0.74
Bricklayers, Stonemasons and Tile Setters (9-51)	409/635	11/15	1.01 (0.44-2.35)							
Bricklayer (construction) (9-51.20)	413/639	6/5	2.12 (0.62-7.22)							
Reinforced Concreters, Cement Finishers and Terrazzo Workers (9-52)	409/640	11/10	1.49 (0.59-3.73)	209/640	5/10	1.34 (0.43-4.16)	159/640	6/10	1.95 (0.61-6.25)	0.65
Carpenters, Joiners and Parquetry Workers (9-54)	407/637	13/13	1.82 (0.81-4.09)	208/637	6/13	1.62 (0.56-4.64)	159/637	6/13	2.22 (0.79-6.28)	0.68
Construction joiner (9-54.20)	413/640	7/10	1.15 (0.42-3.14)							
Plasterers (9-55)	415/641	5/9	0.84 (0.26-2.67)							
Construction Workers N.E.C. (9-59)	410/637	10/13	1.31 (0.56-3.09)							
Material Handling and Related Equipment Operators, Dockers and Freight Handlers (9-7)	374/594	46/56	1.28 (0.83-1.97)	194/594	20/56	1.01 (0.57-1.79)	145/594	20/56	1.50 (0.83-2.69)	0.35
Dockers and Freight Handlers (9-71)	385/611	35/39	1.30 (0.79-2.15)	201/611	13/39	0.82 (0.41-1.64)	148/611	17/39	1.83 (0.95-3.52)	0.10
Warehouse porter (9-71.45)	400/626	20/24	1.17 (0.62-2.23)	205/626	9/24	0.83 (0.36-1.91)	156/626	9/24	1.77 (0.76-4.12)	0.21
Machine packer (9-71.55)	411/642	9/8	1.35 (0.49-3.67)							
Material Handling Equipment Operators N.E.C (9-79)	410/636	10/14	1.27 (0.54-2.97)							
Lifting-truck operator (9-79.20)	411/636	9/14	1.12 (0.47-2.68)							
Transport Equipment Operators (9-8)	386/599	34/51	1.03 (0.64-1.65)	195/599	19/51	0.96 (0.53-1.73)	150/599	15/51	1.38 (0.73-2.62)	0.42

Motor-vehical drivers (9-85)	388/602	32/48	1.02 (0.62-1.66)	196/602	18/48	0.95 (0.52-1.73)	151/602	14/48	1.37 (0.71-2.63)	0.42
Lorry and van driver (local transport) (9-85.50)	402/625	18/25	1.08 (0.57-2.07)	207/625	7/25	0.65 (0.27-1.59)	154/625	11/25	2.11 (0.96-4.64)	0.05
Other motor-vehicle drivers (9-85.90)	414/642	6/8	1.00 (0.31-3.18)							
Laboureres N.E.C (9-9)	408/634	12/16	1.07 (0.48-2.38)							

Abbreviations - OR: odds ratio; 95% CI: confidence interval of 95%; ISCO: International Standard Classification of Occupations; Ca/Co (ever)/(never): Cases/controls ever/never employed; N.E.C: Not Elsewhere Classified.

[a] Total number of subjects presented for each code can vary due to the management of codes with missing digits. Subjects with a single job coded as missing data, were excluded from the analyses (N=33).

[b] Estimates obtained comparing TGCT cases to group A and group B controls combined and adjusted for sibship size, being born from multiple pregnancy, personal history of testicular trauma, family history of TGCT and family history of cryptorchidism. Analysis was restricted to subjects with no missing data for the adjustment variables (N=12). Results presented if a job was held by more than five cases and five controls (grey line if less).

[c] 219 cases of seminoma TGCT were present in the TESTIS study.

[d] 191 cases of non-seminoma TGCT were present in the TESTIS study.

[e] P-value for heterogeneity derived from the Likelihood Ratio Test, comparing seminoma versus non-seminoma tumours.

Table S2. Odds ratios and 95% confidence intervals for TGCT associated with industries, overall and cording to histological subtypes, TESTIS study – *full table*.

**Industry description (NAF-99 code)** [a]	**All TGCT cases**			**Seminomas** [c]			**Non-seminomas** [d]			**P-HET**[e]
	Ca/Co (never)	*Ca/Co (ever)*	**OR (95% IC)** [b]	*Ca/Co (never)*	*Ca/Co (ever)*	**OR (95% IC)** [b]	*Ca/Co (never)*	*Ca/Co (ever)*	**OR (95% IC)** [b]	
**Agriculture, hunting and forestry (01, 02)**	386/615	33/34	1.43 (0.85-2.41)	198/612	16/34	1.32 (0.69-2.54)	148/612	16/34	1.64 (0.83-3.25)	0.65
Agriculture, hunting and related service activities (01)	387/618	32/31	1.56 (0.91-2.67)	198/615	16/31	1.48 (0.76-2.88)	149/615	15/31	1.79 (0.88-3.64)	0.70
Culture (01.1)	404/636	15/13	1.82 (0.84-3.97)	206/633	8/13	1.67 (0.66-4.28)	158/633	6/13	2.11 (0.72-6.17)	0.75
Farming of animals (01.2)	408/643	11/6	2.62 (0.92-7.49)							
Farming of cattle, dairy farming (01.2A)	412/643	7/5	2.03 (0.60-6.87)							
Services related to agriculture and landscape management (01.4)	411/639	8/10	0.98 (0.36-2.64)							
Creation and maintenance of ornamental plantations (01.4B)	412/640	7/9	1.06 (0.37-3.01)							
**Manufacturing (15 to 37)**	288/458	131/191	1.10 (0.83-1.46)	147/456	67/190	1.13 (0.79-1.61)	116/456	48/190	1.00 (0.67-1.50)	0.66
Manufacture of food products and beverages (15)	393/615	26/34	1.24 (0.72-2.16)	203/612	11/34	0.96 (0.46-1.99)	150/612	14/34	1.76 (0.88-3.53)	0.24
Production, processing and preserving of meat and meat products (15.1)	410/643	9/6	2.12 (0.71-6.31)							
Industrial preparation of meat products (15.1E)	413/644	6/5	1.77 (0.52-6.07)							
Manufacture of other food products (15.8)	408/630	11/19	1.00 (0.45-2.22)							
Publishing, printing and reproduction of recorded media (22)	412/642	7/7	1.67 (0.56-4.99)							
Manufacture of chemicals and chemical products (24)	405/632	14/17	1.17 (0.54-2.50)	207/629	7/17	1.08 (0.43-2.75)	159/629	5/17	1.05 (0.35-3.14)	0.96
Manufacture of pharmaceuticals, medicinal chemicals and botanical products (24.4)	410/641	9/8	1.26 (0.45-3.52)							
Manufacture of rubber and plastic products (25)	413/642	6/7	1.26 (0.38-4.19)							
Manufacture of other non-metallic mineral products (26)	408/641	11/8	1.83 (0.71-4.76)							

Manufacture of basic metals (27)	409/640	10/9	2.19 (0.81-5.96)							
Metalworking (28)	403/619	16/30	0.84 (0.44-1.61)	206/617	8/29	0.82 (0.35-1.89)	157/584	7/29	0.92 (0.37-2.34)	0.85
Metal processing, general mechanics (28.5)	413/639	6/10	0.81 (0.27-2.38)							
Manufacture of machinery and equipment (29)	407/617	12/32	0.56 (0.27-1.14)	208/614	6/32	0.60 (0.24-1.52)	158/614	6/32	0.76 (0.29-2.00)	0.74
Manufacture of general-purpose machinery (29.2)	413/633	6/16	0.62 (0.23-1.73)							
Manufacture of machinery and electrical (31)	407/638	12/11	1.59 (0.66-3.81)							
Manufacture of radio, television and communication equipment (32)	414/638	5/11	0.83 (0.27-2.50)							
Manufacture of medical, precision, optical and watchmaking instruments (33)	413/638	6/11	0.84 (0.30-2.38)							
Manufacture of measuring and control instruments (33.2)	413/643	6/6	1.42 (0.44-4.63)							
Manufacture of motor vehicles, trailers and semi-trailers (34)	404/623	15/26	1.00 (0.50-2.01)	205/620	9/26	1.31 (0.58-2.95)	159/620	5/26	0.79 (0.25-2.46)	0.48
Construction of motor vehicles (34.1)	411/635	8/14	1.00 (0.40-2.48)							
Manufacture of other transport equipment (35)	403/630	16/19	1.36 (0.67-2.77)							
Manufacture of aircraft and spacecraft (35.3)	409/633	10/16	0.92 (0.39-2.17)							
Manufacture of furniture; manufacturing n.e.c (36)	412/640	7/9	1.18 (0.42-3.32)							
Manufacture of furniture (36.1)	414/645	5/4	2.24 (0.57-8.80)							
**Electricity, gas and water supply (40, 41)**	404/630	15/19	1.12 (0.53-2.36)	209/627	5/19	0.80 (0.28-2.28)	158/627	6/19	0.88 (0.32-2.40)	0.90
Electricity, gas, steam and hot water supply (40)	407/632	12/17	0.98 (0.44-2.19)	209/629	5/17	0.85 (0.29-2.46)	159/629	5/17	0.75 (0.25-2.25)	0.88
Production and distribution of electricity (40.1)	410/633	9/16	0.75 (0.31-1.83)							
Electricity generation and distribution (40.1Z)	410/633	9/16	0.75 (0.31-1.83)							
**Construction (45)**	333/541	86/108	1.27 (0.91-1.77)	178/540	36/106	0.94 (0.61-1.47)	127/540	37/106	1.41 (0.89-2.22)	0.22
Site preparation (45.1)	413/635	5/8	0.76 (0.23-2.50)							

Construction of building and civil engineering works (45.2)	392/614	26/30	1.38 (0.78-2.43)	206/612	7/30	0.61 (0.26-1.47)	149/612	15/30	1.80 (0.88-3.66)	0.06
General masonry work (45.2V)	406/631	10/8	1.76 (0.66-4.69)							
Installation work (45.3)	371/602	47/41	1.99 (1.25-3.15)	193/600	20/41	1.52 (0.84-2.75)	148/600	16/41	1.59 (0.83-3.03)	0.92
Electrical installation work (45.3A)	389/617	29/26	1.98 (1.12-3.50)	199/615	14/26	1.66 (0.82-3.38)	154/615	10/26	1.96 (0.89-4.34)	0.76
Water and gas installation (45.3E)	411/635	7/8	1.19 (0.41-3.44)							
Installation of heating and air conditioning equipment (45.3F)	404/635	14/8	2.95 (1.18-7.39)							
Finishing work (45.4)	398/609	21/34	0.87 (0.48-1.56)	204/608	10/33	0.82 (0.38-1.78)	154/608	10/33	1.15 (0.53-2.51)	0.54
Wood and plastic joinery (45.4C)	409/632	10/11	1.34 (0.54-3.30)							
**Trade, repair of motor vehicles and household goods (50 to 52)**	293/495	126/154	1.42 (1.06-1.90)	146/492	68/154	1.34 (0.94-1.92)	118/492	46/154	1.41 (0.93-2.14)	0.86
Motor trade and repair (50)	395/622	24/27	1.40 (0.78-2.52)	198/619	16/27	1.59 (0.81-3.12)	157/619	7/27	1.16 (0.47-2.86)	0.57
Motor vehicle trade (50.1)	413/641	6/8	1.48 (0.50-4.41)							
Motor vehicle trade (50.1Z)	413/641	6/8	1.48 (0.50-4.41)							
Maintenance and repair of motor vehicles (50.2)	406/634	13/15	1.40 (0.65-3.03)							
Wholesale trade and commercial intermediaries (51)	375/596	44/53	1.35 (0.87-2.10)	191/593	23/53	1.25 (0.73-2.16)	146/593	18/53	1.44 (0.79-2.65)	0.73
Wholesale intermediaries (51.1)	414/643	5/6	1.22 (0.34-4.30)							
Wholesale of food products (51.3)	414/641	5/8	0.87 (0.26-2.91)							
Wholesale of non-food consumer goods (51.4)	409/639	10/10	1.75 (0.70-4.36)							
Wholesale of non-agricultural intermediate products (51.5)	413/638	6/11	0.86 (0.30-2.41)							
Wholesale of industrial equipment (51.6)	402/630	17/19	1.59 (0.79-3.19)	205/627	9/19	1.66 (0.72-3.80)	156/627	8/19	1.96 (0.80-4.85)	0.79
Retail trade and repair of household goods (52)	345/560	74/89	1.38 (0.97-1.96)	177/557	37/89	1.18 (0.76-1.82)	136/557	28/83	1.46 (0.90-2.39)	0.52
Retail sale in non-specialised shops (52.1)	388/621	31/28	1.91 (1.11-3.28)	202/618	12/28	1.29 (0.63-2.66)	148/618	16/28	3.00 (1.51-5.98)	0.10
Supermarkets (52.1D)	389/626	30/23	2.28 (1.28-4.06)	202/623	12/23	1.64 (0.78-3.46)	149/623	15/23	3.20 (1.55-6.60)	0.21
Food retail in specialized shops (52.2)	409/642	10/7	2.89 (1.03-8.14)							
Other retail sale in specialised shops (52.4)	385/608	34/41	1.12 (0.69-1.84)	195/605	19/41	1.13 (0.63-2.04)	153/605	11/41	1.03 (0.50-2.12)	0.83
Retail sale of sports and recreation	408/642	11/7	1.79 (0.66-4.80)							

equipment (52.4W)										
Retail trade (non-store) (52.6)	414/635	5/14	0.67 (0.23-1.94)							
**Hotels and restaurants (55)**	368/580	51/69	1.23 (0.82-1.84)	190/578	24/68	1.07 (0.63-1.79)	141/578	23/68	1.34 (0.79-2.29)	0.56
Hotels (55.1)	406/636	13/13	1.58 (0.70-3.54)							
Tourist hotels with restaurant (55.1A)	407/637	12/11	1.63 (0.69-3.86)							
Restaurants (55.3)	391/604	28/45	1.06 (0.63-1.75)	200/602	14/44	1.05 (0.55-2.01)	151/602	13/44	1.11 (0.56-2.18)	0.91
Traditional food service (55.3A)	398/624	21/23	1.61 (0.85-3.05)	205/621	9/23	1.25 (0.55-2.85)	152/621	12/23	1.90 (0.89-4.08)	0.46
Fast food restaurants (55.3B)	408/624	11/23	0.74 (0.34-1.58)							
Coffee shops (55.4)	413/644	6/5	2.36 (0.70-7.92)							
Canteens and caterers (55.5)	412/642	7/7	1.32 (0.43-4.08)							
**Transport and communications (60 to 64)**	374/555	45/94	0.74 (0.50-1.09)	184/552	30/94	0.94 (0.59-1.50)	152/552	12/94	0.49 (0.26-0.94)	0.10
Land transport (60)	396/607	23/42	0.88 (0.51-1.51)	200/604	14/42	1.09 (0.56-2.09)	156/604	8/42	0.77 (0.35-1.70)	0.51
Urban and road transport (60.2)	399/618	19/28	1.04 (0.56-1.93)	203/615	10/28	1.08 (0.49-2.35)	156/615	8/28	1.11 (0.48-2.54)	0.96
Intercity road freight transport (60.2M)	411/635	7/10	0.93 (0.33-2.58)							
Auxiliary transport services (63)	405/627	14/22	1.03 (0.51-2.10)							
Post and telecommunications (64)	407/618	12/31	0.58 (0.29-1.16)							
Telecommunications (64.2)	411/626	8/23	0.55 (0.24-1.29)							
Other telecommunications activities (64.2B)	411/629	8/20	0.65 (0.28-1.54)							
**Financial activities (65 to 67)**	399/614	20/35	1.05 (0.58-1.88)	204/611	10/35	0.95 (0.45-2.03)	154/611	10/35	1.72 (0.80-3.69)	0.29
Financial intermediation (65)	403/621	16/28	0.96 (0.50-1.84)	205/618	9/28	0.97 (0.43-2.17)	157/618	7/28	1.38 (0.57-3.35)	0.57
**Real estate, rental and business services (70 to 74)**	318/453	101/196	0.77 (0.57-1.03)	152/450	62/196	0.91 (0.63-1.31)	136/450	28/196	0.51 (0.32-0.81)	0.05
Real estate (70)	414/639	5/10	0.90 (0.29-2.72)							
Rental without operator (71)	414/643	5/6	1.43 (0.41-4.96)							
Computer and related activities (72)	386/589	33/60	0.88 (0.55-1.40)	195/586	19/60	0.93 (0.52-1.65)	151/586	13/60	0.85 (0.44-1.65)	0.84
Hardware consultancy (72.1)	389/605	12/17	1.09 (0.50-2.37)	196/602	7/17	1.06 (0.40-2.78)	153/602	5/17	1.22 (0.42-3.55)	0.85
Software development (72.2)	396/606	5/17	0.51 (0.18-1.42)							
Research and development (73)	406/614	13/35	0.59 (0.30-1.17)	206/611	8/35	0.67 (0.30-1.52)	159/611	5/35	0.64 (0.24-1.72)	0.94
Research and experimental development on natural sciences and engineering (73.1)	407/616	12/31	0.60 (0.29-1.23)	207/613	7/31	0.64 (0.27-1.54)	159/613	5/31	0.71 (0.26-1.93)	0.89
Other business activities (74)	357/541	62/108	0.91 (0.64-1.30)	176/538	38/108	1.06 (0.69-1.63)	150/538	14/108	0.50 (0.27-0.92)	0.04
Legal, accounting and management	400/609	19/40	0.78 (0.44-1.39)	206/606	8/40	0.63 (0.28-1.39)	158/606	6/40	0.65 (0.26-1.62)	0.95

consultancy activities (74.1)										
Business and management consulting (74.1G)	406/626	13/23	0.92 (0.45-1.88)	208/623	6/23	0.81 (0.31-2.09)	159/623	5/23	0.89 (0.32-2.50)	0.90
Architectural and engineering activities and related technical consultancy (74.2)	405/625	14/24	0.91 (0.45-1.84)							
Engineering, technical studies (74.2C)	408/628	11/21	0.75 (0.34-1.64)							
Publicity (74.4)	413/639	6/10	1.08 (0.37-3.11)							
Selection and supply of personnel (74.5)	414/640	5/9	0.50 (0.16-1.60)							
Temporary work (74.5B)	414/642	5/7	0.62 (0.18-2.11)							
Investigation and security (74.6)	412/636	7/13	0.91 (0.34-2.40)							
Cleaning activities (74.7)	412/637	7/12	1.00 (0.38-2.64)							
Miscellaneous business activities N.E.C. (74.8)	414/643	5/6	1.51 (0.44-5.22)							
**Public administration (75)**	348/542	71/107	0.98 (0.69-1.38)	180/539	34/107	0.83 (0.52-1.30)	133/539	31/107	1.15 (0.71-1.86)	0.33
General, economic and social administration (75.1)	387/612	32/37	1.33 (0.79-2.21)	200/609	14/37	1.07 (0.54-2.09)	147/609	17/37	1.86 (0.97-3.55)	0.24
General public administration (75.1A)	391/616	28/33	1.37 (0.79-2.36)	202/613	12/33	1.13 (0.55-2.33)	149/613	15/33	1.84 (0.93-3.66)	0.34
Supervision of economic activities (75.1E)	413/644	6/5	1.26 (0.37-4.30)							
Services of public prerogative (75.2)	379/579	40/70	0.81 (0.53-1.25)	192/576	22/70	0.82 (0.47-1.40)	151/576	13/70	0.66 (0.33-1.30)	0.63
Defence (75.2C)	393/597	26/52	0.71 (0.42-1.19)	198/594	16/52	0.83 (0.44-1.54)	155/594	9/52	0.60 (0.27-1.35)	0.54
Civil protection (75.2J)	406/636	13/13	1.28 (0.57-2.90)							
**Education (80)**	324/494	95/155	0.97 (0.72-1.31)	169/491	45/155	0.78 (0.52-1.15)	126/491	38/155	0.99 (0.65-1.51)	0.41
Primary education (80.1)	413/639	6/10	0.88 (0.31-2.52)							
Secondary education (80.2)	353/569	66/80	1.33 (0.92-1.92)	182/566	32/80	1.03 (0.64-1.66)	139/566	25/80	1.27 (0.76-2.13)	0.55
General secondary education (80.2A)	406/624	13/21	0.88 (0.41-1.88)	208/621	6/21	0.50 (0.18-1.41)	159/621	5/21	1.33 (0.46-3.86)	0.20
Technical or professional secondary education (80.2C)	365/586	54/57	1.55 (1.03-2.34)	188/583	26/57	1.31 (0.78-2.21)	143/583	21/57	1.35 (0.77-2.39)	0.94
Higher education (80.3)	394/587	25/62	0.71 (0.43-1.17)	202/584	12/62	0.65 (0.34-1.26)	153/584	11/62	0.77 (0.38-1.56)	0.73
**Health and social work (85)**	388/565	31/84	0.57 (0.37-0.89)	196/562	18/84	0.64 (0.36-1.11)	151/562	13/84	0.61 (0.32-1.16)	0.92
Human health activities (85.1)	396/585	23/63	0.56 (0.33-0.93)	204/582	10/63	0.45 (0.22-0.93)	151/582	13/63	0.79 (0.41-1.52)	0.26
Hospital activities (85.1A)	400/597	19/51	0.56 (0.32-0.98)	208/594	6/51	0.33 (0.13-0.81)	151/594	13/51	1.01 (0.52-1.96)	0.04
Social action (85.3)	411/624	8/25	0.53 (0.23-1.20)							

**Other community, social and personal services (90 to 93)**	360/578	59/71	1.48 (1.00-2.19)	185/575	29/71	1.23 (0.75-2.01)	137/575	27/71	2.26 (1.32-3.87)	0.10
Sanitation, roads and waste management (90)	412/641	7/8	1.29 (0.45-3.68)							
Association activities (91)	410/643	9/6	2.61 (0.90-7.62)							
Recreational, cultural and sporting activities (92)	375/593	44/56	1.40 (0.91-2.17)	193/590	21/56	1.09 (0.62-1.90)	144/590	20/56	2.07 (1.14-3.76)	0.12
Film and video activities (92.1)	414/640	5/9	1.11 (0.36-3.40)							
Other service activities (92.3)	402/634	17/15	1.92 (0.91-4.02)	204/631	10/15	1.80 (0.76-4.30)	159/631	5/15	1.67 (0.55-5.06)	0.91
Sporting activities (92.6)	407/629	12/20	0.99 (0.46-2.12)							
Management of sports facilities (92.6A)	414/642	5/7	1.06 (0.31-3.63)							
Other sports activities (92.6C)	411/636	8/13	1.07 (0.43-2.66)							
Other recreational activities (92.7)	413/638	6/11	0.88 (0.32-2.44)							
Other recreational activities N.E.C. (92.7C)	413/639	6/10	0.96 (0.34-2.71)							

Abbreviations - OR: odds ratio; 95% CI: confidence interval of 95%; ISCO: International Standard Classification of Occupations; Ca/Co (ever)/(never): Cases/controls ever/never employed; N.E.C: Not Elsewhere Classified.

[a] Total number of subjects presented for each code can vary due to the management of codes with missing digits. Subjects with a single job coded as missing data, were excluded from the analyses (N=33).

[b] Estimates obtained comparing TGCT cases to group A and group B controls combined and adjusted for sibship size, being born from multiple pregnancy, personal history of testicular trauma, family history of TGCT and family history of cryptorchidism. Analysis was restricted to subjects with no missing data for the adjustment variables (N=12). Results presented if a job was held by more than five cases and five controls.

Table S3. Odds ratios and 95% confidence intervals for TGCT associated with industries, according to employment duration (< 2 vs. ≥ 2 years), TESTIS study.

**Industry description (NAF-99 code)** [a]	*Ca/Co (never)*	*Ca/Co < 2 YEARS*	**OR1 (95% IC)** [b]	*Ca/Co ≥ 2 YEARS*	**OR2 (95% IC)** [b]	**P-trend** [c]
**Agriculture,hunting and forestry (01, 02)**	391/617	9/8	1.81 (0.68-4.81)	24/26	1.38 (0.77-2.48)	0.28
Agriculture, hunting and related service activities (01)	388/620	9/7	1.84 (0.65-5.22)	23/24	1.48 (0.80-2.74)	0.25
**Manufacturing (15 to 37)**	292/460	43/55	1.22 (0.79-1.88)	88/ 136	1.06 (0.77-1.45)	0.66
Metalworking (28)	404/621	10/11	1.48 (0.60-3.65)	6/19	0.47 (0.17-1.26)	0.22
**Electricity, gas and water supply (40, 41)**	409/632	5/7	0.89 (0.26-2.97)	10/12	1.42 (0.59-3.36)	0.73
Production and distribution of electricity, gas and heat (40)	408/634	5/6	0.94 (0.26-3.35)	7/11	1.00 (0.36-2.80)	0.99
**Construction (45)**	334/543	28/38	1.27 (0.74-2.16)	58/70	1.27 (0.86-1.88)	0.38
Construction of building and civil engineering works (45.2)	390/612	9/11	1.62 (0.63-4.15)	17/19	1.27 (0.63-2.56)	0.49
Installation work (45.3)	369/600	18/16	1.91 (0.93-3.95)	29/25	**2.03 (1.15-3.59)**	**0.01**
Electrical installation work (45.3A)	389/617	9/10	1.69 (0.65-4.39)	20/16	**2.11 (1.06-4.19)**	0.06
Finishing work (45.4)	396/607	8/13	0.90 (0.35-2.31)	13/21	0.86 (0.41-1.79)	0.90
**Trade, auto repair and household goods (50, 51, 52)**	293/495	39/50	1.32 (0.83-2.10)	87/ 104	**1.45 (1.04-2.02)**	0.07
Wholesale trade and commercial intermediaries (51)	376/598	17/17	1.58 (0.76-3.30)	27/36	1.25 (0.73-2.13)	0.36
Retail trade and repair of household goods (52)	346/562	29/40	1.15 (0.69-1.93)	45/49	**1.58 (1.02-2.47)**	0.59
Retail sale in non-specialised shops (52.1)	386/619	18/13	**2.26 (1.07-4.76)**	13/15	1.60 (0.73-3.49)	**0.03**
Supermarkets (52.1D)	412/638	18/10	**2.80 (1.25-6.28)**	12/13	1.68 (0.74-3.83)	**0.02**
**Hotels and restaurants (55)**	369/582	17/26	1.00 (0.52-1.94)	34/43	1.38 (0.85-2.26)	0.43
**Transport and communication (60 to 64)**	374/555	10/33	**0.47 (0.22-0.98)**	35/61	0.89 (0.57-1.41)	0.12
**Real estate, rental and business services (70 to 74)**	318/453	25/53	0.69 (0.41-1.17)	76/ 143	0.81 (0.58-1.13)	0.22
Other business activities (74)	358/543	25/32	1.14 (0.64-2.01)	37/76	0.81 (0.53-1.25)	0.56
**Public administration (75)**	352/553	20/27	1.14 (0.61-2.12)	48/70	1.01 (0.67-1.53)	0.92
**Education (80)**	381/579	14/23	1.02 (0.51-2.04)	25/49	0.77 (0.45-1.32)	0.64
**Health and social work (85)**	389/567	8/23	0.57 (0.25-1.31)	23/61	0.57 (0.34-0.96)	0.05
Hospital activities (85.1A)	423/609	5/9	0.92 (0.30-2.85)	14/42	0.48 (0.25-0.90)	0.08
**Other community, social and personal services (90 to 93)**	3/578	15/18	1.47 (0.71-3.03)	44/53	1.51 (0.97-2.37)	0.12
Recreational, cultural and sporting activities (92)	376/595	8/11	1.33 (0.51-3.47)	36/45	1.42 (0.88-2.30)	0.31

Abbreviations - OR: odds ratio; 95% CI: confidence interval of 95%; ISCO: International Standard Classification of Occupations; Ca/Co (ever)/(never): Cases/controls ever/never employed; N.E.C: Not Elsewhere Classified.

[a] Total number of subjects presented for each code can vary due to the management of codes with missing digits. Subjects with a single job coded as missing data, were excluded from the analyses (N=33).

[b] Estimates obtained comparing TGCT cases to group A and group B controls combined and adjusted for sibship size, being born from multiple pregnancy, personal history of testicular trauma, family history of TGCT and family history of cryptorchidism. Analysis was restricted to subjects with no missing data for the adjustment variables (N=12). Results presented if a job was held by more than five cases and five controls.

Table S4. Odds ratios and 95% confidence intervals for TGCT associated with occupations, excluding cases with personal history of cryptorchidism, TESTIS study.

**Occupation description (ISCO-68)** [a]	*Ca/Co (never)*	*Ca/Co (ever)*	**OR (95% IC)** [b,c]
**Professional, Technical and Related Workers (0/1)**	228/355	153/295	0.85 (0.65-1.12)
Architects, engineers and related technicians (0-2/0-3)	334/567	47/83	0.98 (0.65-1.47)
Mechanical Engineers (0-24)	373/634	7/16	0.73 (0.27-1.96)
Draughtsmen (0-32)	369/639	11/11	1.80 (0.73-4.44)
Civil Engineering Technicians (0-33)	373/637	7/13	0.92 (0.35-2.44)
Clerk of works (0-33.40)	373/639	7/11	1.00 (0.32-2.71)
Engineering Technicians N.E.C. (0-39)	373/632	7/18	0.68 (0.27-1.70)
Other engineering technicians (0-39.90)	375/640	5/10	0.79 (0.25-2.47)
Life scientists and related technicians (0-5)	371/639	10/11	1.46 (0.58-3.68)
Doctors, dentists, veterinarians and similar workers (06/07)	367/605	14/45	0.50 (0.26-0.94)
Professional Nurses (0-71)	375/637	6/13	0.71 (0.26-1.93)
Professional nurse (general) (0-71.10)	375/639	6/11	0.81 (0.29-2.25)
Statisticians, mathematicians, systems analysts and related technicians (0-8)	346/597	35/53	1.18 (0.73-1.90)
Systems Analysts (0-83)	348/603	33/47	1.29 (0.78-2.11)
Accountants (1-1)	371/640	10/10	1.78 (0.71-4.50)
Accountants (1-10)	371/640	10/10	1.78 (0.71-4.50)
Auditor (1-10.20)	371/641	10/9	2.02 (0.78-5.23)
Teachers (1-3)	355/593	26/57	0.80 (0.48-1.33)
University and Higher Education Teachers (1-31)	374/628	7/22	0.69 (0.29-1.67)
Secondary Education Teachers (1-32)	364/622	17/28	1.05 (0.54-2.01)
Other secondary education teachers (1-32.90)	370/632	8/15	0.93 (0.37-2.29)
Authors, Journalists and Related Writers (1-5)	376/644	5/6	1.64 (0.49-5.55)
Authors, Journalists and Related Writers N.E.C (1-59)	376/645	5/5	1.89 (0.53-6.73)
Painters, Photographers and Related Creative Artists (1-6)	375/644	6/6	1.86 (0.57-6.07)
Composers and Performing Artists (1-7)	373/643	8/7	2.06 (0.71-5.93)
Athletes, Sportsmen and Related Workers (1-8)	375/633	6/17	0.66 (0.26-1.73)
Athletes, Sportsmen and Related Workers (1-80)	375/633	6/17	0.66 (0.26-1.73)
Sports coach (1-80.30)	375/634	6/16	0.72 (0.28-1.90)
Professional, Technical and Related Workers N.E.C (1-9)	369/619	12/31	0.66 (0.33-1.33)
Social Workers (1-93)	372/626	9/24	0.61 (0.27-1.37)

Culture centre worker (1-93.30)	373/630	7/12	0.88 (0.33-2.37)
**Administrative and Managerial Workers (2)**	330/544	51/106	0.87 (0.59-1.28)
Managers (2-1)	330/545	51/105	0.88 (0.60-1.29)
General managers (2-11)	375/622	6/22	0.54 (0.21-1.38)
Managers N.E.C (2-19)	335/563	46/82	1.05 (0.69-1.58)
Sales manager (except wholesale and retail trade) (2-19.30)	373/636	8/8	1.68 (0.60-4.69)
Budgeting and accounting manager (2-19.50)	375/629	6/13	0.91 (0.33-2.51)
Other managers (2-19.90)	348/581	33/61	0.96 (0.60-1.54)
**Clerical and Related Workers (3)**	309/523	72/127	0.94 (0.67-1.31)
Bookkeepers, cashiers and related workers (3-3)	372/629	9/21	0.60 (0.26-1.37)
Bookkeepers and Cashiers (3-31)	376/642	5/8	0.75 (0.23-2.44)
Bookkeepers, Cashiers and Related Workers N.E.C (3-39)	376/637	5/13	0.62 (0.21-1.79)
Computing Machine Operators (3-4)	372/634	9/16	0.95 (0.40-2.24)
Clerical and Related Workers N.E.C (3-9)	327/569	54/81	1.14 (0.78-1.67)
Stock clerks (3-91)	350/609	31/41	1.23 (0.75-2.03)
Dispatching and receiving clerk (3-91.20)	366/627	15/23	1.12 (0.56-2.21)
Storeroom clerk (3-91.40)	366/632	15/18	1.28 (0.62-2.65)
Correspondence and Reporting Clerks (3-93)	369/627	12/23	0.96 (0.47-1.99)
Office clerk (general) (3-93.10)	372/633	9/17	0.94 (0.41-2.18)
Receptionists and Travel Agency Clerks (3-94)	368/628	13/22	0.94 (0.46-1.95)
Receptionist (general) (3-94.10)	372/640	9/10	1.40 (0.55-3.59)
**Sales Workers (4)**	296/538	85/112	1.38 (0.99-1.93)
Working Proprietors (Wholesale and Retail Trade) (4-1)	374/642	7/8	1.82 (0.62-5.35)
Technical Salesmen, Commercial Travellers and Manufacturers' Agents (4-3)	349/597	32/53	1.02 (0.63-1.63)
Technical Salesmen and Service Advisers (4-31)	349/599	32/51	1.05 (0.65-1.69)
Insurance, Real Estate, Securities and Business Services Salesmen and Auctioneers (4-4)	375/638	6/12	0.88 (0.32-2.45)
Insurance, Real Estate and Securities Salesmen (4-41)	375/639	6/11	0.95 (0.34-2.67)
Salesmen, Shop Assistants and Related Workers (4-5)	329/597	52/53	1.73 (1.13-2.65)
Salesmen, Shop Assistants and Demonstrators (4-51)	329/603	52/47	1.92 (1.24-2.96)
Retail trade salesman (4-51.30)	353/622	28/28	1.67 (0.96-2.92)
Other salesmen, shop assistants and demonstrators (4-51.90)	351/631	30/19	2.64 (1.43-4.86)
**Service Workers (5)**	293/510	88/140	1.13 (0.83-1.56)

Cooks, Waiters, Bartenders and Related Workers (5-3)	346/592	35/58	1.13 (0.72-1.79)
Cooks pressers (5-31)	363/616	18/34	0.94 (0.52-1.72)
Cook, except private service (5-31.30)	373/636	8/14	1.14 (0.46-2.81)
Other cooks (5-31.90)	369/624	12/26	0.78 (0.38-1.60)
Waiters, Bartenders and Related Workers Workers (5-32)	356/621	25/29	1.74 (0.98-3.10)
Waiter, general (5-32.10)	362/631	19/19	1.99 (1.01-3.94)
Bartender (5-32.50)	371/640	10/10	2.22 (0.89-5.56)
Building Caretakers, Charworkers, Cleaners and Related Workers (5-5)	348/594	33/56	1.04 (0.66-1.67)
Charworkers, Cleaners and Related Workers (5-52)	348/594	33/56	1.04 (0.66-1.67)
Charworker (5-52.20)	351/595	30/55	0.96 (0.60-1.56)
Protective Service Workers (5-8)	347/602	34/48	1.20 (0.74-1.94)
Fire-Fighters (5-81)	369/639	12/11	1.69 (0.72-3.99)
Fire-fighter (general) (5-81.10)	370/639	11/11	1.50 (0.62-3.60)
Protective Service Workers N.E.C (5-89)	357/617	24/33	1.26 (0.72-2.23)
Watchman (5-89.40)	370/634	11/16	1.21 (0.53-2.73)
Other protective service workers (5-89.90)	366/635	15/15	1.70 (0.79-3.66)
Service Workers N.E.C (5-9)	373/628	8/22	0.62 (0.27-1.44)
Other Service Workers (5-99)	373/631	8/19	0.70 (0.30-1.67)
**Agricultural, Animal Husbandry and Forestry Workers (6)**	342/611	39/39	1.60 (1.03-2.59)
Farmers (6-1)	372/641	9/9	1.91 (0.72-5.05)
Specialised Farmers (6-12)	376/644	5/6	1.63 (0.48-5.61)
Agricultural and Animal Husbandry Workers (6-2)	350/619	31/31	1.56 (0.91-2.67)
Nursery Workers and Gardeners (6-27)	365/635	16/15	1.54 (0.74-3.23)
Gardener (6-27.40)	365/636	16/14	1.62 (0.76-3.43)
**Production and related workers, transport equipment operators and labourers (7/8/9)**	198/385	183/265	1.33 (1.02-1.75)
Production Supervisors and General Foremen (7-0)	363/620	18/30	1.15 (0.62-2.13)
Production Supervisors and General Foremen (7-00)	363/620	18/30	1.15 (0.62-2.13)
Production supervisor and general foreman (general) (7-00.10)	369/638	12/12	2.14 (0.94-4.89)
Food and Beverage Processers (7-7)	373/627	8/23	0.65 (0.28-1.52)
Blacksmiths, Toolmakers and Machine Tool Operators (8-3)	370/633	11/17	0.78 (0.34-1.80)
Machinery Fitters,Machine Assemblers and Precision- Instrument Makers (except Electrical) (8-4)	329/593	52/57	1.71 (1.12-2.61)

Machinery Fitters and Machine Assemblers (8-41)	367/639	14/11	2.49 (1.07-5.75)
Refrigeration and air-conditioning plan installer and mechanic (8-41.80)	371/642	10/8	2.29 (0.86-6.14)
Motor-Vehicle Mechanics (8-43)	368/631	13/19	1.19 (0.56-2.51)
Automobile mechanic (8-43.20)	375/639	6/11	1.02 (0.36-2.87)
Motor-truck mechanic (8-43.30)	374/644	7/6	1.78 (0.57-5.57)
Machinery Fitters, Machine Assemblers and Precision-Instrument Makers (except Electrical) N.E.C (8-49)	353/618	28/32	1.55 (0.89-2.69)
Agricultural machinery mechanic (8-49.55)	376/645	5/5	1.75 (0.48-6.36)
Plant maintenance mechanic (8-49.70)	367/633	14/17	1.35 (0.64-2.87)
Electrical Fitters and Related Electrical and Electronics Workers (8-5)	339/613	42/37	2.00 (1.24-3.24)
Electrical Wiremén (8-55)	355/626	26/24	1.81 (1.30-3.28)
Building electrician (8-55.20)	361/629	20/21	1.61 (0.84-3.08)
Plumbers, Welders, Sheet-Metal and Structural Metal Preparers and Erectors (8-7)	351/620	30/30	1.69 (0.97-2.93)
Plumbers and Pipe Fitters (8-71)	368/638	13/12	1.69 (0.74-3.87)
Plumber (general) (8-71.05)	368/641	13/9	2.35 (0.96-5.73)
Welders and Flame-Cutters (8-72)	369/638	12/12	1.61 (0.68-3.80)
Sheet-Metal Workers (8-73)	376/642	5/8	1.11 (0.34-3.61)
Rubber and Plastics Product Makers (9-0)	375/645	6/5	2.44 (0.71-8.36)
Painters (9-3)	374/636	7/14	0.62 (0.24-1.61)
Bricklayers, Carpenters and Other Construction Workers (9-5)	348/601	33/49	1.12 (0.69-1.83)
Bricklayers, Stonemasons and Tile Setters (9-51)	371/635	10/15	0.99 (0.41-2.36)
Bricklayer (construction) (9-51.20)	375/639	5/5	1.91 (0.52-7.06)
Reinforced Concreters, Cement Finishers and Terrazzo Workers (9-52)	371/640	10/10	1.41 (0.55-3.62)
Carpenters, Joiners and Parquetry Workers (9-54)	371/637	10/13	1.57 (0.66-3.73)
Construction joiner (9-54.20)	376/640	5/10	0.95 (0.31-2.88)
Plasterers (9-55)	376/641	5/9	0.94 (0.30-2.98)
Construction Workers N.E.C (9-59)	373/637	8/13	1.22 (0.49-3.05)
Material Handling and Related Equipment Operators, Dockers and Freight Handlers (9-7)	341/594	40/56	1.26 (0.81-1.97)
Dockers and Freight Handlers (9-71)	349/611	32/39	1.36 (0.81-2.26)
Warehouse porter (9-71.45)	362/626	19/24	1.28 (0.67-2.45)
Machine packer (9-71.55)	372/642	9/8	1.48 (0.55-4.00)
Material Handling Equipment Operators N.E.C (9-79)	373/636	8/14	1.12 (0.45-2.78)

Lifting-truck operator (9-79.20)	374/636	7/14	0.95 (0.37-2.47)
Transport Equipment Operators (9-8)	350/599	31/51	1.06 (0.65-1.73)
Motor-vehical drivers (9-85)	352/602	29/48	1.04 (0.63-1.72)
Lorry and van driver (local transport) (9-85.50)	365/625	16/25	1.05 (0.54-2.06)
Other motor-vehicle drivers (9-85.90)	376/642	5/8	1.03 (0.31-3.37)
Laboureres N.E.C (9-9)	372/634	9/16	0.84 (0.35-2.02)

Abbreviations - OR: odds ratio; 95% CI: confidence interval of 95%; ISCO: International Standard Classification of Occupations; Ca/Co (ever)/(never): Cases/controls ever/never employed; N.E.C: Not Elsewhere Classified.

[a] Total number of subjects presented for each code can vary due to the management of codes with missing digits. Subjects with a single job coded as missing data, were excluded from the analyses (N=33).

[b] Estimates obtained comparing TGCT cases to group A and group B controls combined and adjusted for sibship size, being born from multiple pregnancy, personal history of testicular trauma, family history of TGCT and family history of cryptorchidism. Analysis was restricted to subjects with no missing data for the adjustment variables (N=12). Results presented if a job was held by more than five cases and five controls.

[c] 40 cases with personal history of cryptorchidism were excluded from the analyses.

Table S5. Odds ratios and 95% confidence intervals for TGCT associated with industries, excluding cases with personal history of cryptorchidism, TESTIS study.

Industry description (NAF-99 code) [a]	*Ca/Co (never)*	*Ca/Co (ever)*	OR (95% IC) [b,c]
**Agriculture,hunting and forestry (01, 02)**	351/615	29/34	1.37 (0.80-2.36)
Agriculture, hunting, related services (01)	352/618	28/31	1.50 (0.86-2.61)
Culture (01.1)	368/636	12/13	1.62 (0.71-3.70)
Livestock (01.2)	370/643	10/6	2.65 (0.91-7.76)
Cattle breeding (01.2A)	374/643	6/5	1.88 (0.53-6.66)
Services related to agriculture and landscape management (01.4)	372/639	8/10	1.06 (0.39-2.88)
Creation and maintenance of ornamental plantations (01.4B)	373/640	7/9	1.17 (0.41-3.32)
**Manufacture industry (15 to 37)**	261/458	119/191	1.11 (0.83-1.49)
Food industry (15)	358/615	22/34	1.18 (0.66-2.10)
Meat industry (15.1)	374/643	6/6	1.49 (0.45-4.94)
Other food industries (15.8)	370/630	10/19	1.04 (0.46-2.35)
Publishing, printing, reproduction (22)	375/642	5/7	1.27 (0.38-4.22)
Chemical industry (24)	366/632	14/17	1.35 (0.63-2.90)
Pharmaceutical industry (24.4)	371/641	9/8	1.39 (0.50-3.87)
Rubber and plastics industry (25)	375/642	5/7	1.33 (0.39-4.56)
Manufacture of other non-metallic mineral products (26)	370/641	10/8	1.80 (0.67-4.83)
Metallurgy (27)	371/640	9/9	2.21 (0.80-6.12)
Metalworking (28)	367/619	13/30	0.73 (0.36-1.48)
Metal processing, general mechanics (28.5)	375/639	5/10	0.78 (0.25-2.44)
Manufacture of machinery and equipment (29)	368/617	12/32	0.62 (0.30-1.26)
Manufacture of general-purpose machinery (29.2)	374/633	6/16	0.68 (0.25-1.86)
Manufacture of machinery and electrical (31)	368/638	12/11	1.80 (0.75-4.34)
Manufacture of medical, precision, optical and watchmaking instruments (33)	375/638	5/11	0.81 (0.27-2.42)
Manufacture of measuring and control instruments (33.2)	375/643	5/6	1.39 (0.41-4.77)
Automobile industry (34)	366/623	14/26	1.00 (0.49-2.04)
Construction of motor vehicles (34.1)	372/635	8/14	1.08 (0.44-2.69)
Manufacture of other transport equipment (35)	364/630	16/19	1.56 (0.76-3.19)
Aeronautical and space construction (35.3)	370/633	10/16	1.09 (0.46-2.56)

Manufacture of furniture; miscellaneous industries (36)	374/640	6/9	1.00 (0.34-2.94)
**Production and distribution of electricity, gas and water (40, 41)**	365/630	15/19	1.23 (0.59-2.58)
Production and distribution of electricity, gas and heat (40)	368/632	12/17	1.07 (0.48-2.39)
Electricity generation and distribution (40.1)	371/633	9/16	0.82 (0.34-2.00)
**Construction (45)**	301/541	79/108	1.27 (0.90-1.78)
Construction of building and civil engineering works (45.2)	356/614	23/30	1.29 (0.72-2.32)
General masonry work (45.2V)	368/631	9/8	1.73 (0.63-4.74)
Installation work (45.3)	334/602	45/41	2.03 (1.28-3.23)
Electrical installation work (45.3A)	350/617	29/26	2.16 (1.22-3.81)
Water and gas installation (45.3E)	372/635	7/8	1.40 (0.48-4.07)
Installation of heating and air conditioning equipment (45.3F)	367/635	12/8	2.48 (0.96-6.38)
Finishing work (45.4)	362/609	18/34	0.81 (0.43-1.49)
Wood and plastic joinery (45.4C)	372/632	8/11	1.18 (0.45-3.08)
**Trade, repair of motor vehicles and household goods (50 to 52)**	262/495	118/154	1.47 (1.09-1.98)
Motor trade and repair (50)	358/622	22/27	1.37 (0.75-2.49)
Motor vehicle trade (50.1)	374/641	6/8	1.57 (0.52-4.68)
Maintenance and repair of motor vehicles (50.2)	369/634	11/15	1.25 (0.56-2.83)
Wholesale trade and commercial intermediaries (51)	339/596	41/53	1.38 (0.88-2.16)
Wholesale of non-food consumer goods (51.4)	371/639	9/10	1.76 (0.69-4.45)
Wholesale of non-agricultural intermediate products (51.5)	374/638	6/11	0.88 (0.31-2.48)
Wholesale of industrial equipment (51.6)	363/630	17/19	1.70 (0.85-3.41)
Retail trade and repair of household goods (52)	310/560	70/89	1.45 (1.02-2.07)
Retail sale in non-specialised shops (52.1)	351/621	29/28	1.93 (1.11-3.36)
Supermarkets (52.1D)	352/626	28/23	2.32 (1.29-4.17)
Food retail in specialized shops (52.2)	370/642	10/7	3.10 (1.11-8.67)
Other retail sale in specialised shops (52.4)	348/608	32/41	1.17 (0.71-1.93)
Retail sale of sports and recreation equipment (52.4W)	370/642	10/7	1.93 (0.71-5.27)
Retail trade (non-store) (52.6)	379/644	5/14	0.75 (0.26-2.16)
**Hotels and restaurants (55)**	336/580	44/69	1.22 (0.80-1.85)

Hotels (55.1)	371/636	9/13	1.25 (0.51-3.06)
Tourist hotels with restaurant (55.1A)	372/637	8/11	1.25 (0.48-3.26)
Restaurants (55.3)	354/604	26/46	1.10 (0.65-1.84)
Traditional food service (55.3A)	361/624	19/23	1.63 (0.85-3.13)
Fast food restaurants (55.3B)	369/624	11/23	0.82 (0.39-1.76)
Coffee shops (55.4)	374/644	6/5	2.65 (0.78-8.97)
Canteens and caterers (55.5)	375/642	5/7	1.22 (0.37-4.06)
**Transport and communications (60 to 64)**	338/555	42/94	0.76 (0.50-1.13)
Land transport (60)	358/607	22/42	0.93 (0.54-1.62)
Urban and road transport (60.2)	361/618	18/28	1.09 (0.58-2.04)
Intercity road freight transport (60.2M)	373/635	6/10	0.90 (0.31-2.63)
Auxiliary transport services (63)	367/627	13/22	1.05 (0.51-2.19)
Post and telecommunications (64)	369/618	11/31	0.56 (0.27-1.16)
Telecommunications (64.2)	373/626	7/23	0.51 (0.21-1.25)
Other telecommunications activities (64.2B)	373/629	7/20	0.61 (0.25-1.51)
**Financial activities (65 to 67)**	361/614	19/35	1.07 (0.59-1.94)
Financial intermediation (65)	365/621	15/28	0.96 (0.50-1.87)
**Real estate, rental and business services (70 to 74)**	288/453	92/196	0.79 (0.58-1.06)
Real estate (70)	375/639	5/10	0.98 (0.33-2.97)
IT activities (72)	349/589	31/60	0.90 (0.56-1.44)
IT systems consulting (72.1)	352/605	12/17	1.20 (0.55-2.62)
Software development (72.2)	359/606	5/17	0.55 (0.20-1.53)
Research and development (73)	368/614	12/35	0.63 (0.31-1.28)
Research and development in the physical and natural sciences (73.1)	369/616	11/31	0.65 (0.31-1.35)
Services provided mainly to businesses (74)	323/541	57/108	0.93 (0.65-1.34)
Legal, accounting and management consultancy activities (74.1)	362/609	18/40	0.78 (0.43-1.40)
Business and management consulting (74.1G)	368/626	12/23	0.90 (0.44-1.88)
Activités d'architecture et d'ingénierie (74.2)	366/625	14/24	1.03 (0.51-2.09)
Engineering, technical studies (74.2C)	369/628	11/21	0.85 (0.39-1.87)
Publicity (74.4)	375/6398	5/10	0.97 (0.32-2.98)
Investigation and security (74.6)	373/636	7/13	1.03 (0.39-2.74)

Cleaning activities (74.7)	373/637	7/12	1.14 (0.43-3.04)
Various services provided mainly to businesses (74.8)	375/643	5/6	1.62 (0.47-5.59)
**Public administration (75)**	315/542	65/107	0.98 (0.69-1.41)
General, economic and social administration (75.1)	351/612	29/37	1.28 (0.76-2.17)
General public administration (75.1A)	355/616	25/33	1.30 (0.74-2.29)
Supervision of economic activities (75.1E)	375/644	5/5	1.14 (0.31-4.13)
Services of public prerogative (75.2)	343/579	37/70	0.84 (0.54-1.31)
Defence (75.2C)	356/597	24/52	0.73 (0.43-1.25)
Civil protection (75.2J)	368/636	12/13	1.35 (0.59-3.11)
**Education (80)**	298/494	82/155	0.89 (0.65-1.22)
Primary education (80.1)	375/639	5/10	0.77 (0.25-2.37)
Secondary education (80.2)	324/569	56/80	1.21 (0.82-1.77)
General secondary education (80.2A)	368/624	12/21	0.93 (0.43-2.00)
Technical or professional secondary education (80.2C)	335/586	45/57	1.36 (0.89-2.09)
Higher education (80.3)	358/587	22/62	0.67 (0.40-1.14)
**Health and social work (85)**	354/565	26/84	0.52 (0.32-0.83)
Activities for human health (85.1)	362/585	18/63	0.47 (0.27-0.82)
Hospital activities (85.1A)	364/597	16/51	0.51 (0.29-0.93)
Social action (85.3)	372/624	8/25	0.57 (0.25-1.30)
**Other community, social and personal services (90 to 93)**	328/578	52/71	1.45 (0.97-2.17)
Sanitation, roads and waste management (90)	374/641	6/8	1.26 (0.42-3.76)
Association activities (91)	372/643	8/6	2.73 (0.91-8.20)
Recreational, cultural and sporting activities (92)	340/593	40/56	1.39 (0.89-2.17)
Other artistic and performing activities (92.3)	364/634	16/15	1.93 (0.91-4.07)
Sports-related activities (92.6)	369/629	11/20	1.03 (0.48-2.23)
Management of sports facilities (92.6A)	375/642	5/7	1.25 (0.37-4.24)
Other sports activities (92.6C)	373/636	7/13	1.06 (0.41-2.70)
Recreational activities (92.7)	374/638	6/11	0.95 (0.34-2.65)
Other recreational activities (92.7C)	374/639	6/10	1.05 (0.37-2.96)

Abbreviations - OR: odds ratio; 95% CI: confidence interval of 95%; ISCO: International Standard Classification of Occupations; Ca/Co (ever)/(never): Cases/controls ever/never employed; N.E.C: Not Elsewhere Classified.

[a] Total number of subjects presented for each code can vary due to the management of codes with missing digits. Subjects with a single job coded as missing data, were excluded from the analyses (N=33).

[b] Estimates obtained comparing TGCT cases to group A and group B controls combined and adjusted for sibship size, being born from multiple pregnancy, personal history of testicular trauma, family history of TGCT and family history of cryptorchidism. Analysis was restricted to subjects with no missing data for the adjustment variables (N=12). Results presented if a job was held by more than five cases and five controls.
[c] 40 cases with personal history of cryptorchidism were excluded from the analyses.

Table S6. Odds ratios and 95% confidence intervals for TGCT associated with occupations, excluding cases not confirmed by pathology reports, TESTIS study.

Occupation description (ISCO-68) [a]	*Ca/Co (never)*	*Ca/Co (ever)*	OR (95% IC) [b,c]
**Professional, Technical and Related Workers (0/1)**	232/355	148/295	0.80 (0.61-1.06)
Architects, engineers and related technicians (0-2/0-3)	336/567	44/83	0.87 (0.57-1.32)
Mechanical Engineers (0-24)	373/634	6/16	0.58 (0.20-1.67)
Draughtsmen (0-32)	369/639	10/11	1.57(0.62-3.99)
Civil Engineering Technicians (0-33)	371/637	8/13	1.02 (0.40-2.62)
Clerk of works (0-33.40)	371/639	8/11	1.12 (0.42-2.93)
Engineering Technicians N.E.C (0-39)	372/632	7/18	0.69 (0.28-1.73)
Other engineering technicians (0-39.90)	374/640	5/10	0.78 (0.25-2.42)
Life scientists and related technicians (0-5)	368/639	12/11	1.54 (0.63-3.75)
Doctors, dentists, veterinarians and similar workers (0-6/0-7)	363/605	17/45	0.64 (0.35-1.16)
Medical Doctors (0-61)	375/634	5/16	0.54 (0.19-1.53)
Professional Nurses (0-71)	374/637	6/13	0.72 (0.27-1.96)
Professional nurse (general) (0-71.10)	374/639	6/11	0.82 (0.29-2.29)
Statisticians, mathematicians, systems analysts and related technicians (0-8)	345/597	35/53	1.26 (0.78-2.04)
Systems Analysts (0-83)	347/603	33/47	1.38 (0.84-2.27)
Accountants (1-1)	373/640	7/10	1.44 (0.53-3.94)
Accountants (1-10)	373/640	7/10	1.44 (0.53-3.94)
Auditor (1-10.20)	373/641	7/9	1.64 (0.58-4.59)
Teachers (1-3)	354/593	26/57	0.81 (0.49-1.35)
University and Higher Education Teachers (1-31)	373/628	7/22	0.70 (0.29-1.68)
Secondary Education Teachers (1-32)	363/622	17/28	1.07 (0.56-2.08)
Other secondary education teachers (1-32.90)	368/632	9/15	1.08 (0.45-2.61)
Painters, Photographers and Related Creative Artists (1-6)	374/644	6/6	1.79 (0.55-5.80)
Composers and Performing Artists (1-7)	374/643	6/7	1.50 (0.48-4.73)
Athletes, Sportsmen and Related Workers (1-8)	374/633	6/17	0.66 (0.25-1.72)
Athletes, Sportsmen and Related Workers (1-80)	374/633	6/17	0.66 (0.25-1.72)
Sports coach (1-80.30)	374/634	6/16	0.73 (0.28-1.91)
Professional, Technical and Related Workers N.E.C (1-9)	369/619	11/31	0.62 (0.30-1.27)
Social Workers (1-93)	372/626	8/24	0.56 (0.24-1.31)
Culture centre worker (1-93.30)	373/630	6/12	0.77 (0.27-2.19)

**Administrative and Managerial Workers (2)**	332/544	48/106	0.85 (0.58-1.27)
Managers (2-1)	332/545	48/105	0.86 (0.58-1.28)
General managers (2-11)	374/622	6/22	0.51 (0.20-1.32)
Managers N.E.C (2-19)	338/563	42/82	1.01 (0.66-1.55)
Sales manager (except wholesale and retail trade) (2-19.30)	373/636	7/8	1.54 (0.53-4.49)
Budgeting and accounting manager (2-19.50)	375/629	5/13	0.82 (0.28-2.41)
Other managers (2-19.90)	348/581	32/61	1.00 (0.62-1.61)
**Clerical and Related Workers (3)**	303/523	77/127	0.97 (0.69-1.35)
Bookkeepers, cashiers and related workers (3-3)	370/629	10/21	0.66 (0.30-1.46)
Bookkeepers and Cashiers (3-31)	375/642	5/8	0.77 (0.24-2.51)
Bookkeepers, Cashiers and Related Workers N.E.C (3-39)	374/637	6/13	0.71 (0.26-1.95)
Computing Machine Operators (3-4)	370/634	10/16	1.01 (0.43-2.36)
Clerical and Related Workers N.E.C (3-9)	322/569	58/81	1.18 (0.81-1.72)
Stock clerks (3-91)	350/609	30/41	1.15 (0.69-1.91)
Dispatching and receiving clerk (3-91.20)	366/627	14/23	1.04 (0.52-2.07)
Storeroom clerk (3-91.40)	365/632	15/18	1.24 (0.60-2.57)
Correspondence and Reporting Clerks (3-93)	366/627	14/23	1.07 (0.53-2.15)
Office clerk (general) (3-93.10)	368/633	12/17	1.21 (0.56-2.61)
Receptionists and Travel Agency Clerks (3-94)	363/628	17/22	1.23 (0.62-2.43)
Receptionist (general) (3-94.10)	367/640	13/10	2.03 (0.85-4.85)
**Sales Workers (4)**	300/538	80/112	1.25 (0.90-1.75)
Working Proprietors (Wholesale and Retail Trade) (4-1)	374/642	6/8	1.55 (0.50-4.81)
Technical Salesmen, Commercial Travellers and Manufacturers' Agents (4-3)	349/597	31/53	0.98 (0.61-1.58)
Technical Salesmen and Service Advisers (4-31)	349/599	31/51	1.01 (0.63-1.64)
Insurance, Real Estate, Securities and Business Services Salesmen and Auctioneers (4-4)	374/638	6/12	0.87 (0.31-2.43)
Insurance, Real Estate and Securities Salesmen (4-41)	374/639	6/11	0.93 (0.33-2.64)
Salesmen, Shop Assistants and Related Workers (4-5)	332/597	48/53	1.55 (1.01-2.39)
Salesmen, Shop Assistants and Demonstrators (4-51)	332/603	48/47	1.72 (1.11-2.68)
Retail trade salesman (4-51.30)	357/622	23/28	1.33 (0.74-2.39)
Other salesmen, shop assistants and demonstrators (4-51.90)	351/631	29/19	2.56 (1.38-4.75)

**Service Workers (5)**	285/510	95/140	1.21 (0.88-1.64)
Cooks, Waiters, Bartenders and Related Workers (5-3)	338/592	42/58	1.30 (0.84-2.01)
Cooks pressers (5-31)	357/616	23/34	1.15 (0.65-2.04)
Cook, except private service (5-31.30)	366/636	14/14	1.76 (0.80-3.86)
Other cooks (5-31.90)	366/624	14/26	0.87 (0.44-1.75)
Waiters, Bartenders and Related Workers Workers (5-32)	353/621	27/29	1.79 (1.02-3.16)
Waiter, general (5-32.10)	359/631	21/19	2.13 (1.10-4.14)
Bartender (5-32.50)	370/640	10/10	2.13 (0.85-5.32)
Building Caretakers, Charworkers, Cleaners and Related Workers (5-5)	343/594	37/56	1.14 (0.73-1.78)
Charworkers, Cleaners and Related Workers (5-52)	343/594	37/56	1.14 (0.73-1.78)
Charworker (5-52.20)	346/595	34/55	1.06 (0.67-1.69)
Protective Service Workers (5-8)	349/602	31/48	1.10 (0.67-1.80)
Fire-Fighters (5-81)	371/639	9/11	1.22 (0.49-3.07)
Fire-fighter (general) (5-81.10)	372/639	8/11	1.04 (0.40-2.68)
Protective Service Workers N.E.C (5-89)	356/617	24/33	1.28 (0.73-2.26)
Watchman (5-89.40)	369/634	11/16	1.24 (0.55-2.79)
Other protective service workers (5-89.90)	365/635	15/15	1.72 (0.80-3.68)
Service Workers N.E.C (5-9)	372/628	8/22	0.59 (0.25-1.39)
Other Service Workers (5-99)	372/631	8/19	0.66 (0.28-1.58)
**Agricultural, Animal Husbandry and Forestry Workers (6)**	337/611	43/39	1.76 (1.10-2.81)
Farmers (6-1)	370/641	10/9	1.94 (0.75-5.00)
Specialised Farmers (6-12)	374/644	6/6	1.77 (0.54-5.75)
Agricultural and Animal Husbandry Workers (6-2)	346/619	34/31	1.75 (1.03-2.96)
Nursery Workers and Gardeners (6-27)	362/635	18/15	1.79 (0.87-3.69)
Gardener (6-27.40)	363/636	17/14	1.75 (0.83-3.67)
**Production and related workers, transport equipment operators and labourers (7/8/9)**	196/385	184/265	1.30 (0.99-1.71)
Production Supervisors and General Foremen (7-0)	360/620	20/30	1.28 (0.70-2.33)
Production Supervisors and General Foremen (7-00)	360/620	20/30	1.28 (0.70-2.33)
Production supervisor and general foreman (general) (7-00.10)	366/638	14/12	2.54 (1.14-5.66)
Food and Beverage Processers (7-7)	369/627	11/23	0.81 (0.38-1.76)
Blacksmiths, Toolmakers and Machine Tool Operators (8-3)	369/633	11/17	0.80 (0.34-1.86)
Machine-Tool Setter-Operators (8-33)	379/648	5/8	0.73 (0.21-2.54)
Machinery Fitters,Machine Assemblers and Precision- Instrument Makers (8-4)	329/593	51/57	1.58 (1.04-2.41)

Machinery Fitters and Machine Assemblers (8-41)	370/639	10/11	1.73 (0.70-4.30)
Refrigeration and air-conditioning plan installer and mechanic (8-41.80)	374/642	6/8	1.38 (0.45-4.21)
Motor-Vehicle Mechanics (8-43)	366/631	14/19	1.21 (0.58-2.52)
Automobile mechanic (8-43.20)	373/639	7/11	1.08 (0.40-2.89)
Motor-truck mechanic (8-43.30)	373/644	7/6	1.77 (0.56-5.61)
Machinery Fitters, Machine Assemblers and Precision-Instrument Makers (except Electrical) N.E.C (8-49)	350/618	30/32	1.62 (0.94-2.79)
Agricultural machinery mechanic (8-49.55)	375/645	5/5	1.74 (0.48-6.36)
Plant maintenance mechanic (8-49.70)	367/633	13/17	1.26 (0.58-2.72)
Electrical Fitters and Related Electrical and Electronics Workers (8-5)	343/613	37/37	1.67 (1.02-2.74)
Electrical Wiremén (8-55)	357/626	23/24	1.48 (0.80-2.72)
Building electrician (8-55.20)	363/629	17/21	1.25 (0.64-2.47)
Plumbers, Welders, Sheet-Metal and Structural Metal Preparers and Erectors (8-7)	353/620	27/30	1.46 (0.83-2.57)
Plumbers and Pipe Fitters (8-71)	369/638	11/12	1.37 (0.58-3.24)
Plumber (general) (8-71.05)	369/641	11/9	1.89 (0.75-4.74)
Welders and Flame-Cutters (8-72)	369/638	11/12	1.35 (0.56-3.27)
Sheet-Metal Workers (8-73)	375/642	5/8	1.16 (0.36-3.78)
Rubber and Plastics Product Makers (9-0)	375/645	5/5	1.89 (0.53-6.77)
Painters (9-3)	372/636	8/14	0.72 (0.29-1.79)
Painters N.E.C (9-39)	375/642	5/8	0.71 (0.22-2.35)
Bricklayers, Carpenters and Other Construction Workers (9-5)	344/601	36/49	1.27 (0.78-2.05)
Bricklayers, Stonemasons and Tile Setters (9-51)	370/635	10/15	1.00 (0.42-2.39)
Bricklayer (construction) (9-51.20)	373/639	6/5	2.29 (0.67-7.81)
Reinforced Concreters, Cement Finishers and Terrazzo Workers (9-52)	369/640	11/10	1.62 (0.64-4.08)
Carpenters, Joiners and Parquetry Workers (9-54)	368/637	12/13	1.76 (0.77-4.05)
Construction joiner (9-54.20)	373/640	7/10	1.23 (0.45-3.37)
Plasterers (9-55)	375/641	5/9	1.25 (0.38-4.16)
Construction Workers N.E.C (9-59)	371/637	9/13	1.35 (0.55-3.34)
Material Handling and Related Equipment Operators, Dockers and Freight Handlers (9-7)	340/594	40/56	1.21 (0.77-1.90)
Dockers and Freight Handlers (9-71)	350/611	30/39	1.18 (0.70-1.98)
Warehouse porter (9-71.45)	362/626	18/24	1.14 (0.59-2.20)
Machine packer (9-71.55)	372/642	8/8	1.28 (0.46-3.58)

Material Handling Equipment Operators N.E.C (9-79)	371/636	9/14	1.36 (0.56-3.32)
Lifting-truck operator (9-79.20)	372/636	8/14	1.19 (0.47-2.99)
Transport Equipment Operators (9-8)	346/599	34/51	1.15 (0.71-1.85)
Motor-vehical drivers (9-85)	348/602	32/48	1.14 (0.70-1.87)
Lorry and van driver (local transport) (9-85.50)	362/625	18/25	1.15 (0.60-2.21)
Other motor-vehicle drivers (9-85.90)	374/642	6/8	1.10 (0.34-3.51)
Laboureres N.E.C (9-9)	370/634	10/16	0.94 (0.40-2.20)

Abbreviations - OR: odds ratio; 95% CI: confidence interval of 95%; ISCO: International Standard Classification of Occupations; Ca/Co (ever)/(never): Cases/controls ever/never employed; N.E.C: Not Elsewhere Classified.

[a] Total number of subjects presented for each code can vary due to the management of codes with missing digits. Subjects with a single job coded as missing data, were excluded from the analyses (N=33).

[b] Estimates obtained comparing TGCT cases to group A and group B controls combined and adjusted for sibship size, being born from multiple pregnancy, personal history of testicular trauma, family history of TGCT and family history of cryptorchidism. Analysis was restricted to subjects with no missing data for the adjustment variables (N=12). Results presented if a job was held by more than five cases and five controls.

[c] 43 cases not confirmed by pathology reports, were excluded from the analyses.

Table S7. Odds ratios and 95% confidence intervals for TGCT associated with industries, excluding cases not confirmed by pathology reports, TESTIS study.

**Industry description (NAF-99 code) [a]**	*Ca/Co (never)*	*Ca/Co (ever)*	**OR (95% IC) [b,c]**
**Agriculture,hunting and forestry (01, 02)**	347/615	32/34	1.49 (0.88-2.51)
Agriculture, hunting, related services (01)	348/618	31/31	1.62 (0.95-2.79)
Culture (01.1)	365/636	14/13	1.82 (0.82-4.02)
Livestock (01.2)	368/643	11/6	2.73 (0.96-7.75)
Cattle breeding (01.2A)	372/643	7/5	2.12 (0.63-7.08)
Services related to agriculture and landscape management (01.4)	371/639	8/10	1.09 (0.40-2.94)
Creation and maintenance of ornamental plantations (01.4B)	372/640	7/9	1.17 (0.41-3.33)
**Manufacture industry (15 to 37)**	263/458	116/191	1.06 (0.79-1.42)
Food industry (15)	354/615	25/34	1.31 (0.75-2.30)
Meat industry (15.1)	370/643	9/6	2.28 (0.76-6.80)
Industrial preparation of meat products (15.1E)	373/644	6/5	1.90 (0.55-6.53)
Other food industries (15.8)	369/630	10/19	1.03 (0.46-2.33)
Chemical industry (24)	367/632	12/17	1.08 (0.49-2.39)
Pharmaceutical industry (24.4)	371/641	8/8	1.13 (0.39-3.28)
Rubber and plastics industry (25)	374/642	5/7	1.18 (0.33-4.18)
Manufacture of other non-metallic mineral products (26)	369/641	10/8	1.84 (0.70-4.85)
Metallurgy (27)	370/640	9/9	2.16 (0.77-6.03)
Metalworking (28)	364/619	15/30	0.85 (0.43-1.68)
Metal processing, general mechanics (28.5)	373/639	6/10	0.82 (0.28-2.43)
Manufacture of machinery and equipment (29)	367/617	12/32	0.62 (0.30-1.26)
Manufacture of general purpose machinery (29.2)	373/633	6/16	0.71 (0.26-1.96)
Manufacture of machinery and electrical (31)	370/638	9/11	1.34 (0.52-3.43)
Manufacture of medical, precision, optical and watchmaking instruments (33)	374/638	5/11	0.83 (0.28-2.46)
Manufacture of measuring and control instruments (33.2)	374/643	5/6	1.38 (0.40-4.67)
Automobile industry (34)	365/623	14/26	1.03 (0.50-2.11)
Construction of motor vehicles (34.1)	372/635	7/14	0.95 (0.36-2.49)
Manufacture of other transport equipment (35)	366/630	13/19	1.18 (0.55-2.54)
Aeronautical and space construction (35.3)	372/633	7/16	0.68 (0.26-1.80)
Manufacture of furniture; miscellaneous industries (36)	372/640	7/9	1.25 (0.44-3.50)
**Production and distribution of electricity, gas and water (40, 41)**	368/630	11/19	0.89 (0.39-2.00)
Production and distribution of electricity, gas and heat (40)	369/632	10/17	0.89 (0.38-2.09)

Electricity generation and distribution (40.1)	372/633	7/16	0.62 (0.24-1.64)
**Construction (45)**	306/541	73/108	1.14 (0.80-1.61)
Site preparation (45.1)	373/635	5/8	0.83 (0.25-2.73)
Construction of building and civil engineering works (45.2)	356/614	22/30	1.22 (0.67-2.21)
General masonry work (45.2V)	366/631	10/8	1.90 (0.71-5.09)
Installation work (45.3)	342/602	36/41	1.54 (0.94-2.52)
Electrical installation work (45.3A)	354/617	24/26	1.68 (0.92-3.07)
Water and gas installation (45.3E)	372/635	6/8	1.18 (0.39-3.59)
Installation of heating and air conditioning equipment (45.3F)	370/635	8/8	1.59 (0.57-4.45)
Finishing work (45.4)	359/609	20/34	0.93 (0.51-1.70)
Wood and plastic joinery (45.4C)	369/632	10/11	1.42 (0.58-3.51)
**Trade, repair of motor vehicles and household goods (50 to 52)**	265/495	114/154	1.37 (1.02-1.85)
Motor trade and repair (50)	356/622	23/27	1.41 (0.78-2.55)
Motor vehicle trade (50.1)	373/641	6/8	1.61 (0.54-4.83)
Maintenance and repair of motor vehicles (50.2)	367/634	12/15	1.34 (0.60-2.98)
Wholesale trade and commercial intermediaries (51)	338/596	41/53	1.39 (0.88-2.17)
Wholesale intermediaries (51.1)	374/643	5/6	1.30 (0.37-4.57)
Wholesale of non-food consumer goods (51.4)	370/639	9/10	1.76 (0.69-4.49)
Wholesale of non-agricultural intermediate products (51.5)	374/638	5/11	0.79 (0.26-2.36)
Wholesale of industrial equipment (51.6)	362/630	17/19	1.75 (0.87-3.53)
Retail trade and repair of household goods (52)	314/560	65/89	1.31 (0.92-1.89)
Retail sale in non-specialised shops (52.1)	351/621	28/28	1.92 (1.10-3.35)
Supermarkets (52.1D)	352/626	27/23	2.29 (1.27-4.13)
Food retail in specialized shops (52.2)	371/642	8/7	2.55 (0.86-7.53)
Other retail sale in specialised shops (52.4)	349/608	30/41	1.07 (0.64-1.78)
Retail sale of sports and recreation equipment (52.4W)	369/642	10/7	1.78 (0.65-4.85)
**Hotels and restaurants (55)**	332/580	47/69	1.29 (0.85-1.95)
Hotels (55.1)	366/636	13/13	1.70 (0.75-3.83)
Tourist hotels with restaurant (55.1A)	367/637	12/11	1.71 (0.72-4.07)
Restaurants (55.3)	352/604	27/45	1.19 (0.71-1.99)
Traditional food service (55.3A)	358/624	21/23	1.80 (0.95-3.41)
Fast food restaurants (55.3B)	369/624	10/23	0.83 (0.38-1.83)
Coffee shops (55.4)	373/644	6/5	2.50 (0.74-8.37)

**Transport and communications (60 to 64)**	337/555	42/94	0.74 (0.49-1.10)
Land transport (60)	357/607	22/42	0.90 (0.52-1.56)
Urban and road transport (60.2)	360/618	18/28	1.05 (0.56-1.97)
Intercity road freight transport (60.2M)	371/635	7/10	0.99 (0.36-2.77)
Auxiliary transport services (63)	366/627	13/22	1.04 (0.50-2.15)
Post and telecommunications (64)	367/618	12/31	0.60 (0.30-1.24)
Telecommunications (64.2)	371/626	8/23	0.60 (0.26-1.41)
Other telecommunications activities (64.2B)	371/629	8/20	0.71 (0.30-1.67)
**Financial activities (65 to 67)**	359/614	20/35	1.21 (0.67-2.17)
Financial intermediation (65)	363/621	16/28	1.09 (0.57-2.09)
**Real estate, rental and business services (70 to 74)**	288/453	91/196	0.76 (0.56-1.03)
Real estate (70)	374/639	5/10	0.96 (0.31-2.93)
Rental without operator (71)	374/643	5/6	1.53 (0.44-5.33)
IT activities (72)	346/589	33/60	0.98 (0.62-1.57)
IT systems consulting (72.1)	349/605	12/17	1.20 (0.55-2.62)
Software development (72.2)	356/606	5/17	0.56 (0.20-1.56)
Research and development (73)	366/614	13/35	0.66 (0.33-1.30)
Research and development in the physical and natural sciences (73.1)	367/616	12/31	0.67 (0.33-1.37)
Services provided mainly to businesses (74)	327/541	52/108	0.83 (0.57-1.20)
Legal, accounting and management consultancy activities (74.1)	365/609	14/40	0.63 (0.33-1.19)
Business and management consulting (74.1G)	368/626	11/23	0.86 (0.40-1.83)
Activités d'architecture et d'ingénierie (74.2)	367/625	12/24	0.86 (0.41-1.81)
Engineering, technical studies (74.2C)	369/628	10/21	0.74 (0.33-1.66)
Publicity (74.4)	374/639	5/10	1.03 (0.34-3.17)
Investigation and security (74.6)	372/636	7/13	1.00 (0.38-2.66)
Cleaning activities (74.7)	374/637	5/12	0.71 (0.24-2.09)
Various services provided mainly to businesses (74.8)	374/643	5/6	1.83 (0.52-6.35)
**Public administration (75)**	314/542	65/107	0.98 (0.68-1.40)
General, economic and social administration (75.1)	348/612	31/37	1.39 (0.83-2.34)
General public administration (75.1A)	352/616	27/33	1.44 (0.83-2.51)
Supervision of economic activities (75.1E)	373/644	6/5	1.30 (0.38-4.46)
Services of public prerogative (75.2)	344/579	35/70	0.78 (0.49-1.22)
Defence (75.2C)	354/597	25/52	0.77 (0.45-1.30)

Civil protection (75.2J)	370/636	9/13	0.96 (0.39-2.35)
**Education (80)**	295/494	84/155	0.92 (0.67-1.25)
Primary education (80.1)	374/639	5/10	0.74 (0.24-2.28)
Secondary education (80.2)	322/569	57/80	1.21 (0.82-1.77)
General secondary education (80.2A)	368/624	11/21	0.80 (0.36-1.78)
Technical or professional secondary education (80.2C)	332/586	47/57	1.42 (0.93-2.17)
Higher education (80.3)	355/587	24/62	0.76 (0.46-1.26)
**Health and social work (85)**	348/565	31/84	0.63 (0.40-0.99)
Activities for human health (85.1)	356/585	23/63	0.62 (0.37-1.03)
Hospital activities (85.1A)	360/597	19/51	0.62 (0.36-1.10)
Social action (85.3)	371/624	8/25	0.57 (0.25-1.29)
**Other community, social and personal services (90 to 93)**	323/578	56/71	1.53 (1.03-2.28)
Sanitation, roads and waste management (90)	372/641	7/8	1.39 (0.49-3.96)
Association activities (91)	370/643	9/6	2.86 (0.98-8.38)
Recreational, cultural and sporting activities (92)	338/593	41/56	1.42 (0.91-2.21)
Film and video activities (92.1)	374/640	5/9	1.20 (0.39-3.71)
Other artistic and performing activities (92.3)	364/634	15/15	1.73 (0.80-3.73)
Sports-related activities (92.6)	367/629	12/20	1.09 (0.51-2.34)
Management of sports facilities (92.6A)	374/642	5/7	1.16 (0.34-3.97)
Other sports activities (92.6C)	371/636	8/13	1.17 (0.47-2.92)

Abbreviations - OR: odds ratio; 95% CI: confidence interval of 95%; ISCO: International Standard Classification of Occupations; Ca/Co (ever)/(never): Cases/controls ever/never employed; N.E.C: Not Elsewhere Classified.

[a] Total number of subjects presented for each code can vary due to the management of codes with missing digits. Subjects with a single job coded as missing data, were excluded from the analyses (N=33).

[b] Estimates obtained comparing TGCT cases to group A and group B controls combined and adjusted for sibship size, being born from multiple pregnancy, personal history of testicular trauma, family history of TGCT and family history of cryptorchidism. Analysis was restricted to subjects with no missing data for the adjustment variables (N=12). Results presented if a job was held by more than five cases and five controls.

[c] 43 cases not confirmed by pathology reports, were excluded from the analyses.

Table S8. Odds ratios and 95% confidence intervals for TGCT associated with occupations, with additional adjustment for age at index date, TESTIS study.

**Occupation description (ISCO-68)** [a]	*Ca/Co (never)*	*Ca/Co (ever)*	**OR (95% IC)** [b]
**Professional, Technical and Related Workers (0/1)**	254/355	166/295	0.78 (0.60-1.01)
Architects, engineers and related technicians (0-2/0-3)	368/567	52/83	0.88 (0.60-1.29)
Mechanical Engineers (0-24)	412/634	7/16	0.60 (0.23-1.57)
Draughtsmen (0-32)	408/639	11/11	1.49 (0.62-3.58)
Civil Engineering Technicians (0-33)	411/637	8/13	0.88 (0.36-2.19)
Clerk of works (0-33.40)	411/639	8/11	1.05 (0.41-2.69)
Electrical and Electronics Engineering Technicians (0-34)	413/640	6/10	0.66 (0.23-1.94)
Engineering Technicians N.E.C (0-39)	411/632	8/18	0.67 (0.29-1.57)
Other engineering technicians (0-39.90)	413/640	6/10	0.91 (0.33-2.57)
**Life scientists and related technicians (0-5)**	408/639	12/11	1.58 (0.66-3.77)
Medical, dental, veterinary and related workers (0-6/0-7)	403/605	17/45	0.58 (0.33-1.04)
Medical Doctors (0-61)	415/634	5/16	0.52 (0.19-1.45)
Professional Nurses (0-71)	414/637	6/13	0.65 (0.24-1.74)
Professional nurse (general) (0-71.10)	414/639	6/11	0.76 (0.27-2.11)
Statisticians, mathematicians, systems analysts and related technicians (0-8)	384/597	36/53	1.09 (0.68-1.72)
Systems Analysts (0-83)	386/603	34/47	1.21 (0.75-1.95)
Accountants (1-1)	410/640	10/10	1.76 (0.71-4.37)
Accountants (1-10)	410/640	10/10	1.76 (0.71-4.37)
Auditor (1-10.20)	410/641	10/9	1.98 (0.78-5.05)
Teachers (1-3)	391/593	29/57	0.77 (0.47-1.24)
University and Higher Education Teachers (1-31)	413/628	7/22	0.53 (0.22-1.27)
Secondary Education Teachers (1-32)	401/622	19/28	1.02 (0.55-1.89)
Other secondary education teachers (1-32.90)	408/632	9/15	0.95 (0.40-2.24)
Authors, Journalists and Related Writers (1-5)	415/644	5/6	1.23 (0.36-4.15)
Authors, Journalists and Related Writers N.E.C (1-59)	415/645	5/5	1.40 (0.39-5.00)
Painters, Photographers and Related Creative Artists (1-6)	414/644	6/6	1.67 (0.53-5.26)
Composers and Performing Artists (1-7)	412/643	8/7	1.86 (0.66-5.25)
Athletes, Sportsmen and Related Workers (1-8)	414/633	6/17	0.57 (0.22-1.46)
Athletes, Sportsmen and Related Workers (1-80)	414/633	6/17	0.57 (0.22-1.46)
Sports coach (1-80.30)	414/634	6/16	0.62 (0.24-1.60)
Professional, Technical and Related Workers N.E.C (1-9)	408/619	12/31	0.65 (0.33-1.30)

Social Workers (1-93)	411/626	9/24	0.63 (0.29-1.39)
Culture centre worker (1-93.30)	412/630	7/12	0.99 (0.38-2.60)
**Administrative and Managerial Workers (2)**	366/544	54/106	0.85 (0.59-1.23)
Managers (2-1)	366/545	54/105	0.86 (0.59-1.24)
General managers (2-11)	413/622	7/22	0.57 (0.24-1.36)
Managers N.E.C (2-19)	372/563	48/82	1.01 (0.68-1.50)
Sales manager (except wholesale and retail trade) (2-19.30)	412/636	8/8	1.59 (0.58-4.36)
Budgeting and accounting manager (2-19.50)	414/629	6/13	0.84 (0.31-2.26)
Other managers (2-19.90)	385/581	35/61	0.98 (0.62-1.54)
**Clerical and Related Workers (3)**	334/523	86/127	1.01 (0.77-1.44)
Bookkeepers, cashiers and related workers (3-3)	410/629	10/21	0.71 (0.32-1.55)
Bookkeepers and Cashiers (3-31)	415/642	5/8	0.89 (0.29-2.80)
Bookkeepers, Cashiers and Related Workers N.E.C (3-39)	414/637	6/13	0.71 (0.26-1.95)
Computing Machine Operators (3-4)	408/634	12/16	1.20 (0.55-2.59)
Clerical and Related Workers N.E.C (3-9)	355/569	65/81	1.25 (0.87-1.79)
Stock clerks (3-91)	385/609	35/41	1.25 (0.77-2.02)
Dispatching and receiving clerk (3-91.20)	404/627	16/23	1.11 (0.57-2.14)
Storeroom clerk (3-91.40)	403/632	17/18	1.26 (0.63-2.52)
Correspondence and Reporting Clerks (3-93)	405/627	15/23	1.04 (0.53-2.06)
Office clerk (general) (3-93.10)	408/633	12/17	1.14 (0.53-2.45)
Receptionists and Travel Agency Clerks (3-94)	403/628	17/22	1.25 (0.65-2.41)
Receptionist (general) (3-94.10)	407/640	13/10	2.17 (0.93-5.07)
**Sales Workers (4)**	332/538	88/112	1.28 (0.93-1.76)
Working Proprietors (Wholesale and Retail Trade) (4-1)	413/642	7/8	2.01 (0.70-5.77)
Technical Salesmen, Commercial Travellers and Manufacturers' Agents (4-3)	388/597	32/53	0.94 (0.59-1.50)
Technical Salesmen and Service Advisers (4-31)	388/599	32/51	0.98 (0.61-1.56)
Insurance, Real Estate, Securities and Business Services Salesmen and Auctioneers (4-4)	414/638	6/12	0.84 (0.31-2.29)
Insurance, Real Estate and Securities Salesmen (4-41)	414/639	6/11	0.91 (0.33-2.51)
Salesmen, Shop Assistants and Related Workers (4-5)	365/597	55/53	1.65 (1.10-2.49)
Salesmen, Shop Assistants and Demonstrators (4-51)	365/603	55/47	1.85 (1.22-2.83)
Retail trade salesman (4-51.30)	391/622	29/28	1.57 (0.90-2.71)
Other salesmen, shop assistants and demonstrators (4-51.90)	388/631	32/19	2.56 (1.41-4.64)
**Service Workers (5)**	319/510	101/140	1.16 (0.86-1.57)

Cooks, Waiters, Bartenders and Related Workers (5-3)	377/592	43/58	1.19 (0.78-1.82)
Cooks pressers (5-31)	396/616	24/34	1.07 (0.62-1.86)
Cook, except private service (5-31.30)	406/636	14/14	1.50 (0.70-3.24)
Other cooks (5-31.90)	405/624	15/26	0.86 (0.44-1.66)
Waiters, Bartenders and Related Workers Workers (5-32)	393/621	27/29	1.63 (0.94-2.84)
Waiter, general (5-32.10)	399/631	21/19	1.87 (0.98-3.58)
Bartender (5-32.50)	410/640	10/10	1.89 (0.77-4.64)
Building Caretakers, Charworkers, Cleaners and Related Workers (5-5)	382/594	38/56	1.07 (0.69-1.68)
Charworkers, Cleaners and Related Workers (5-52)	382/594	38/56	1.07 (0.69-1.68)
Charworker (5-52.20)	386/595	34/55	0.96 (0.61-1.52)
Protective Service Workers (5-8)	385/602	35/48	1.15 (0.72-1.83)
Fire-Fighters (5-81)	407/639	13/11	1.88 (0.82-4.30)
Fire-fighter (general) (5-81.10)	408/639	12/11	1.69 (0.73-3.92)
Protective Service Workers N.E.C (5-89)	396/617	24/33	1.14 (0.65-1.98)
Watchman (5-89.40)	409/634	11/16	1.05 (0.47-2.34)
Other protective service workers (5-89.90)	405/635	15/15	1.59 (0.75-3.34)
Service Workers N.E.C (5-9)	412/628	8/22	0.53 (0.23-1.22)
Other Service Workers (5-99)	412/631	8/19	0.61 (0.26-1.44)
**Agricultural, animal husbandry and forestry workers, fishermen and hunters (6)**	375/611	45/39	1.82 (1.15-2.89)
Farmers (6-1)	410/641	10/9	1.79 (0.70-4.58)
Specialised Farmers (6-12)	414/644	6/6	1.61 (0.50-5.24)
Agricultural and Animal Husbandry Workers (6-2)	384/619	36/31	1.77 (1.06-2.94)
Nursery Workers and Gardeners (6-27)	401/635	19/15	1.84 (0.91-3.72)
Gardener (6-27.40)	402/636	18/14	1.85 (0.90-3.81)
**Production and Related Workers, Transport Equipment operators and labourers (7/8/9)**	216/385	204/265	1.33 (1.03-1.73)
Production Supervisors and General Foremen (7-0)	399/620	21/30	1.18 (0.66-2.12)
Production Supervisors and General Foremen (7-00)	399/620	21/30	1.18 (0.66-2.12)
Production supervisor and general foreman (general) (7-00.10)	406/638	14/12	2.22 (1.00-4.92)
Food and Beverage Processers (7-7)	409/627	11/23	0.78 (0.37-1.65)
Cabinetmakers and Related Woodworkers (8-1)	415/645	5/5	1.38 (0.38-4.98)
Blacksmiths, Toolmakers and Machine Tool Operators (8-3)	408/633	12/17	0.86 (0.38-1.97)
Machinery Fitters,Machine Assemblers and Precision- Instrument Makers (except Electrical) (8-4)	362/593	58/57	1.56 (1.05-2.33)
Machinery Fitters and Machine Assemblers (8-41)	405/639	15/11	2.04 (0.90-4.61)

Refrigeration and air-conditioning plan installer and mechanic (8-41.80)	409/642	11/8	2.02 (0.78-5.22)
Motor-Vehicle Mechanics (8-43)	406/631	14/19	1.08 (0.53-2.23)
Automobile mechanic (8-43.20)	413/639	7/11	0.92 (0.35-2.47)
Motor-truck mechanic (8-43.30)	413/644	7/6	1.73 (0.56-5.31)
Machinery Fitters, Machine Assemblers and Precision-Instrument Makers (except Electrical) N.E.C (8-49)	388/618	32/32	1.54 (0.91-2.60)
Agricultural machinery mechanic (8-49.55)	415/645	5/5	1.45 (0.41-5.15)
Plant maintenance mechanic (8-49.70)	405/633	15/17	1.35 (0.65-2.81)
Electrical Fitters and Related Electrical and Electronics Workers (8-5)	378/613	42/37	1.73 (1.08-2.77)
Electrical Wiremén (8-55)	394/626	26/24	1.60 (0.89-2.86)
Building electrician (8-55.20)	400/629	20/21	1.41 (0.74-2.67)
Plumbers, Welders, Sheet-Metal and Structural Metal Preparers and Erectors (8-7)	388/620	32/30	1.80 (1.06-3.04)
Plumbers and Pipe Fitters (8-71)	406/638	14/12	1.90 (0.85-4.22)
Plumber (general) (8-71.05)	406/641	14/9	2.54 (1.07-6.04)
Welders and Flame-Cutters (8-72)	408/638	12/12	1.61 (0.71-3.66)
Sheet-Metal Workers (8-73)	414/642	6/8	1.27 (0.43-3.81)
Rubber and Plastics Product Makers (9-0)	414/645	6/5	2.04 (0.61-6.77)
Painters (9-3)	412/636	8/14	0.87 (0.35-2.12)
Painters N.E.C (9-39)	415/642	5/8	1.00 (0.32-3.15)
Bricklayers, Carpenters and Other Construction Workers (9-5)	380/601	40/49	1.28 (0.82-2.01)
Bricklayers, Stonemasons and Tile Setters (9-51)	409/635	11/15	1.13 (0.50-2.53)
Bricklayer (construction) (9-51.20)	413/639	6/5	2.12 (0.62-7.24)
Reinforced Concreters, Cement Finishers and Terrazzo Workers (9-52)	409/640	11/10	1.70 (0.70-4.14)
Carpenters, Joiners and Parquetry Workers (9-54)	407/637	13/13	1.58 (0.72-3.46)
Construction joiner (9-54.20)	413/640	7/10	1.06 (0.40-2.86)
Plasterers (9-55)	415/641	5/9	0.85 (0.28-2.63)
Construction Workers N.E.C (9-59)	410/637	10/13	1.18 (0.50-2.78)
Material Handling and Related Equipment Operators, Dockers and Freight Handlers (9-7)	374/594	46/56	1.28 (0.84-1.97)
Dockers and Freight Handlers (9-71)	385/611	35/39	1.35 (0.83-2.21)
Warehouse porter (9-71.45)	400/626	20/24	1.27 (0.68-2.38)
Machine packer (9-71.55)	411/642	9/8	1.51 (0.57-4.04)
Material Handling Equipment Operators N.E.C (9-79)	410/636	10/14	1.18 (0.51-2.72)
Lifting-truck operator (9-79.20)	411/636	9/14	1.06 (0.45-2.50)

Transport Equipment Operators (9-8)	386/599	34/51	1.01 (0.64-1.62)
Motor-vehical drivers (9-85)	388/602	32/48	1.01 (0.62-1.63)
Lorry and van driver (local transport) (9-85.50)	402/625	18/25	1.07 (0.57-2.01)
Other motor-vehicle drivers (9-85.90)	414/642	6/8	1.14 (0.37-3.47)
Laboureres N.E.C (9-9)	408/634	12/16	1.10 (0.54-2.57)

Abbreviations - OR: odds ratio; 95% CI: confidence interval of 95%; ISCO: International Standard Classification of Occupations; Ca/Co (ever)/(never): Cases/controls ever/never employed; N.E.C: Not Elsewhere Classified.

[a] Total number of subjects presented for each code can vary due to the management of codes with missing digits. Subjects with a single job coded as missing data, were excluded from the analyses (N=33).

[b] Estimates obtained comparing TGCT cases to group A and group B controls combined and adjusted for sibship size, being born from multiple pregnancy, personal history of testicular trauma, family history of TGCT, family history of cryptorchidism and age at index date. Analysis was restricted to subjects with no missing data for the adjustment variables (N=12). Results presented if a job was held by more than five cases and five controls.

Table S9. Odds ratios and 95% confidence intervals for TGCT associated with industries, with additional adjustment for age at index date, TESTIS study.

Industry description (NAF-99 code) [a]	*Ca/Co (never)*	*Ca/Co (ever)*	OR (95% IC) [b]
**Agriculture,hunting and forestry (01, 02)**	386/615	33/34	1.46 (0.87-2.43)
Agriculture, hunting, related services (01)	387/618	32/31	1.58 (0.93-2.67)
Culture (01.1)	404/636	15/13	1.81 (0.84-3.93)
Livestock (01.2)	408/643	11/6	2.78 (1.00-7.75)
Cattle breeding (01.2A)	412/643	7/5	2.09 (0.64-6.83)
Services related to agriculture and landscape management (01.4)	411/639	8/10	1.00 (0.38-2.66)
Creation and maintenance of ornamental plantations (01.4B)	412/640	7/9	1.06 (0.38-2.98)
**Manufacture industry (15 to 37)**	288/458	131/191	1.18 (0.75-1.85)
Food industry (15)	393/615	26/34	1.26 (0.74-2.17)
Meat industry (15.1)	410/643	9/6	2.48 (0.85-7.25)
Industrial preparation of meat products (15.1E)	413/644	6/5	1.88 (0.55-6.46)
Other food industries (15.8)	408/630	11/19	0.95 (0.44-2.04)
Publishing, printing, reproduction (22)	412/642	7/7	1.69 (0.58-4.96)
Chemical industry (24)	405/632	14/17	1.19 (0.56-2.51)
Pharmaceutical industry (24.4)	410/641	9/8	1.45 (0.53-3.95)
Rubber and plastics industry (25)	413/642	6/7	1.26 (0.40-3.94)
Manufacture of other non-metallic mineral products (26)	408/641	11/8	1.93 (0.76-4.91)
Metallurgy (27)	409/640	10/9	2.24 (0.87-5.81)
Metalworking (28)	403/619	16/30	0.84 (0.44-1.59)
Metal processing, general mechanics (28.5)	413/639	6/10	0.83 (0.29-2.37)
Manufacture of machinery and equipment (29)	407/617	12/32	0.55 (0.27-1.11)
Manufacture of general-purpose machinery (29.2)	413/633	6/16	0.60 (0.22-1.61)
Manufacture of machinery and electrical (31)	407/638	12/11	1.39 (0.58-3.30)
Manufacture of radio, television and communication equipment (32)	414/638	5/11	0.72 (0.24-2.12)
Manufacture of medical, precision, optical and watchmaking instruments (33)	413/638	6/11	0.82 (0.29-2.27)
Manufacture of measuring and control instruments (33.2)	413/643	6/6	1.50 (0.47-4.79)
Automobile industry (34)	404/623	15/26	0.97 (0.50-1.90)
Construction of motor vehicles (34.1)	411/635	8/14	0.95 (0.39-2.35)
Manufacture of other transport equipment (35)	403/630	16/19	1.30 (0.65-2.62)
Aeronautical and space construction (35.3)	409/633	10/16	0.89 (0.39-2.04)
Manufacture of furniture; miscellaneous industries (36)	412/640	7/9	1.10 (0.40-3.03)

**Electricity,gas and water supply (40, 41)**	404/630	15/19	1.10 (0.54-2.24)
Production and distribution of electricity, gas and heat (40)	407/632	12/17	1.00 (0.46-2.17)
Electricity generation and distribution (40.1)	410/633	9/16	0.80 (0.34-1.88)
**Construction (45)**	333/541	86/108	1.28 (0.92-1.76)
Site preparation (45.1)	413/635	5/8	0.94 (0.30-2.99)
Construction of building and civil engineering works (45.2)	392/614	26/30	1.40 (0.81-2.44)
General masonry work (45.2V)	406/631	10/8	1.99 (0.76-5.19)
Installation work (45.3)	371/602	47/41	1.83 (1.17-2.87)
Electrical installation work (45.3A)	389/617	29/26	1.74 (1.00-3.05)
Water and gas installation (45.3E)	411/635	7/8	1.25 (0.44-3.60)
Installation of heating and air conditioning equipment (45.3F)	404/635	14/8	2.87 (1.17-7.09)
Finishing work (45.4)	398/609	21/34	0.92 (0.52-1.63)
Wood and plastic joinery (45.4C)	409/632	10/11	1.26 (0.52-3.05)
**Trade, repair of motor vehicles and household goods (50 to 52)**	293/495	126/154	1.39 (1.05-1.85)
Motor trade and repair (50)	395/622	24/27	1.32 (0.74-2.36)
Motor vehicle trade (50.1)	413/641	6/8	1.31 (0.44-3.90)
Maintenance and repair of motor vehicles (50.2)	406/634	13/15	1.29 (0.59-2.78)
Wholesale trade and commercial intermediaries (51)	375/596	44/53	1.32 (0.86-2.04)
Wholesale intermediaries (51.1)	414/643	5/6	1.07 (0.31-3.71)
Wholesale of food products (51.3)	414/641	5/8	0.88 (0.27-2.82)
Wholesale of non-food consumer goods (51.4)	409/639	10/10	1.74 (0.68-4.23)
Wholesale of non-agricultural intermediate products (51.5)	413/638	6/11	0.99 (0.36-2.73)
Wholesale of industrial equipment (51.6)	402/630	17/19	1.47 (0.74-2.92)
Retail trade and repair of household goods (52)	345/560	74/89	1.38 (0.98-1.95)
Retail sale in non-specialised shops (52.1)	388/621	31/28	1.88 (1.10-3.22)
Supermarkets (52.1D)	389/626	30/23	2.20 (1.25-3.89)
Food retail in specialized shops (52.2)	409/642	10/7	2.40 (0.88-6.51)
Other retail sale in specialised shops (52.4)	385/608	34/41	1.20 (0.74-1.96)
Retail sale of sports and recreation equipment (52.4W)	408/642	11/7	2.25 (0.83-6.09)
Non-store retail trade (52.6)	414/635	5/14	0.66 (0.23-1.87)
**Hotels and restaurants (55)**	368/580	51/69	1.18 (0.80-1.76)
Hotels (55.1)	406/636	13/13	1.59 (0.71-3.56)
Tourist hotels with restaurant (55.1A)	407/637	12/11	1.70 (0.72-4.01)

Restaurants (55.3)	391/604	28/45	0.99 (0.60-1.64)
Traditional food service (55.3A)	398/624	21/23	1.45 (0.78-2.71)
Fast food restaurants (55.3B)	408/624	11/23	0.72 (0.34-1.51)
Coffee shops (55.4)	413/644	6/5	2.27 (0.67-7.66)
Canteens and caterers (55.5)	412/642	7/7	1.29 (0.43-3.84)
**Transport,storage and communication (60 to 64)**	374/555	45/94	0.72 (0.49-1.07)
Land transport (60)	396/607	23/42	0.87 (0.51-1.50)
Urban and road transport (60.2)	399/618	19/28	1.05 (0.57-1.93)
Intercity road freight transport (60.2M)	411/635	7/10	1.06 (0.39-2.87)
Auxiliary transport services (63)	405/627	14/22	1.01 (0.50-2.05)
Post and telecommunications (64)	407/618	12/31	0.57 (0.28-1.14)
Telecommunications (64.2)	411/626	8/23	0.52 (0.22-1.20)
Other telecommunications activities (64.2B)	411/629	8/20	0.62 (0.26-1.45)
**Financial intermediation (65 to 67)**	399/614	20/35	0.98 (0.55-1.76)
Financial intermediation (65)	403/621	16/28	0.97 (0.51-1.85)
**Real estate,renting and business activities (70 to 74)**	318/453	101/196	0.76 (0.57-1.01)
Real estate (70)	414/639	5/10	0.92 (0.31-2.79)
Rental without operator (71)	414/643	5/6	1.34 (0.40-4.53)
IT activities (72)	386/589	33/60	0.85 (0.54-1.33)
IT systems consulting (72.1)	389/605	12/17	1.09 (0.50-2.36)
Software development (72.2)	396/606	5/17	0.49 (0.18-1.34)
Research and development (73)	406/614	13/35	0.57 (0.29-1.11)
Research and development in the physical and natural sciences (73.1)	407/616	12/31	0.58 (0.29-1.16)
Services provided mainly to businesses (74)	357/541	62/108	0.92 (0.65-1.31)
Legal, accounting and management consultancy activities (74.1)	400/609	19/40	0.81 (0.46-1.43)
Business and management consulting (74.1G)	406/626	13/23	0.98 (0.49-1.98)
Activités d'architecture et d'ingénierie (74.2)	405/625	14/24	0.87 (0.44-1.74)
Engineering, technical studies (74.2C)	408/628	11/21	0.74 (0.34-1.60)
Publicity (74.4)	413/639	6/10	0.99 (0.34-2.84)
Selection and supply of personnel (74.5)	414/640	5/9	0.60 (0.19-1.94)
Temporary work (74.5B)	414/642	5/7	0.71 (0.21-2.42)
Investigation and security (74.6)	412/636	7/13	0.88 (0.33-2.30)
Cleaning activities (74.7)	412/637	7/12	0.96 (0.37-2.51)

Various services provided mainly to businesses (74.8)	414/643	5/6	1.50 (0.45-5.02)
**Public administration (75)**	348/542	71/107	1.05 (0.75-1.48)
General, economic and social administration (75.1)	387/612	32/37	1.33 (0.80-2.22)
General public administration (75.1A)	391/616	28/33	1.45 (0.85-2.47)
Supervision of economic activities (75.1E)	413/644	6/5	1.78 (0.53-6.01)
Services of public prerogative (75.2)	379/579	40/70	0.86 (0.56-1.32)
Defence (75.2C)	393/597	26/52	0.75 (0.45-1.24)
Civil protection (75.2J)	406/636	13/13	1.50 (0.67-3.35)
**Education (80)**	324/494	95/155	0.93 (0.69-1.26)
Primary education (80.1)	413/639	6/10	0.91 (0.32-2.58)
Secondary education (80.2)	353/569	66/80	1.31 (0.91-1.89)
General secondary education (80.2A)	406/624	13/21	0.85 (0.41-1.77)
Technical or professional secondary education (80.2C)	365/586	54/57	1.54 (1.03-2.32)
Higher education (80.3)	394/587	25/62	0.64 (0.39-1.04)
**Health and social work (85)**	388/565	31/84	0.57 (0.37-0.89)
Activities for human health (85.1)	396/585	23/63	0.56 (0.34-0.93)
Hospital activities (85.1A)	400/597	19/51	0.55 (0.32-0.96)
Social action (85.3)	411/624	8/25	0.54 (0.24-1.22)
**Other community, social and personal services (90 to 93)**	360/578	59/71	1.43 (0.98-2.09)
Sanitation, roads and waste management (90)	412/641	7/8	1.33 (0.47-3.76)
Association activities (91)	410/643	9/6	2.59 (0.90-7.49)
Recreational, cultural and sporting activities (92)	375/593	44/56	1.35 (0.88-2.07)
Film and video activities (92.1)	414/640	5/9	0.90 (0.29-2.77)
Other artistic and performing activities (92.3)	402/634	17/15	1.90 (0.93-3.91)
Sports-related activities (92.6)	407/629	12/20	0.94 (0.44-1.98)
Management of sports facilities (92.6A)	414/642	5/7	1.04 (0.31-3.50)
Other sports activities (92.6C)	411/636	8/13	1.01 (0.41-2.49)
Recreational activities (92.7)	413/638	6/11	0.94 (0.34-2.58)
Other recreational activities (92.7C)	413/639	6/10	1.05 (0.37-2.95)

Abbreviations - OR: odds ratio; 95% CI: confidence interval of 95%; ISCO: International Standard Classification of Occupations; Ca/Co (ever)/(never): Cases/controls ever/never employed; N.E.C: Not Elsewhere Classified.

[a] Total number of subjects presented for each code can vary due to the management of codes with missing digits. Subjects with a single job coded as missing data, were excluded from the analyses (N=33).

[b] Estimates obtained comparing TGCT cases to group A and group B controls combined and adjusted for sibship size, being born from multiple pregnancy, personal history of testicular trauma, family history of TGCT, family history of cryptorchidism and age at index date. Analysis was restricted to subjects with no missing data for the adjustment variables (N=12). Results presented if a job was held by more than five cases and five control.
